# Stabilization of the Active Ruthenium Oxycarbonate Phase for Low-Temperature CO_2_ Methanation

**DOI:** 10.1021/acscatal.3c05679

**Published:** 2024-03-06

**Authors:** Carmen Tébar-Soler, Vlad Martin Diaconescu, Laura Simonelli, Alexander Missyul, Virginia Perez-Dieste, Ignacio Villar-García, Daviel Gómez, Jean-Blaise Brubach, Pascale Roy, Avelino Corma, Patricia Concepción

**Affiliations:** † Instituto de Tecnología Química, Universitat Politècnica de València-Consejo Superior de Investigaciones Científicas (UPV-CSIC), Avenida de los Naranjos s/n, 46022 Valencia, Spain; ‡ CELLS - ALBA Synchrotron Radiation Facility, Carrer de la Llum 2-26, 08290 Cerdanyola del Vallès, Spain; § Synchrotron SOLEIL, AILES beamline, L’Orme des Merisiers, 91190 Saint Aubin, France

**Keywords:** ruthenium, methane, CO_2_, operando spectroscopy, interstitial carbon

## Abstract

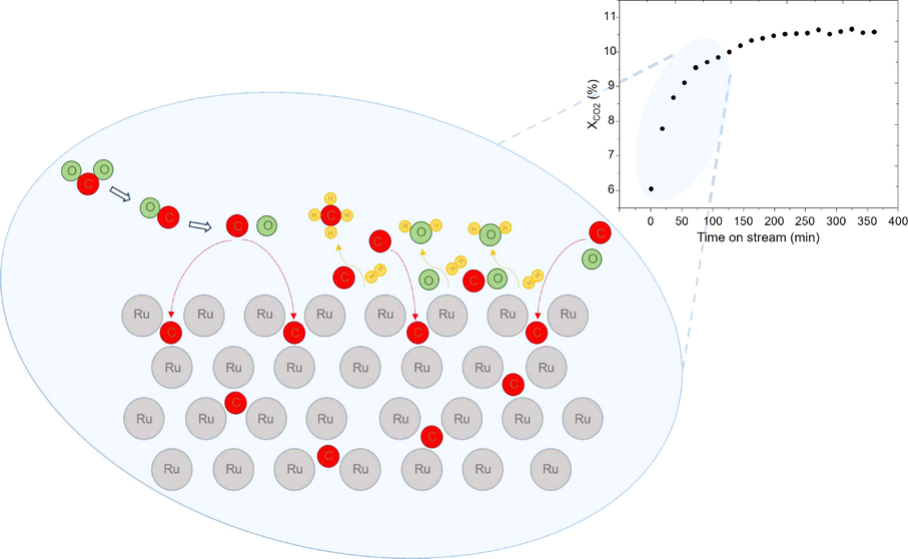

Interstitial carbon-doped RuO_2_ catalyst with the newly reported ruthenium oxycarbonate phase is a key component for low-temperature CO_2_ methanation. However, a crucial factor is the stability of interstitial carbon atoms, which can cause catalyst deactivation when removed during the reaction. In this work, the stabilization mechanism of the ruthenium oxycarbonate active phase under reaction conditions is studied by combining advanced operando spectroscopic tools with catalytic studies. Three sequential processes: carbon diffusion, metal oxide reduction, and decomposition of the oxycarbonate phase and their influence by the reaction conditions, are discussed. We present how the reaction variables and catalyst composition can promote carbon diffusion, stabilizing the oxycarbonate catalytically active phase under steady-state reaction conditions and maintaining catalyst activity and stability over long operation times. In addition, insights into the reaction mechanism and a detailed analysis of the catalyst composition that identifies an adequate balance between the two phases, i.e., ruthenium oxycarbonate and ruthenium metal, are provided to ensure an optimum catalytic behavior.

## Introduction

The CO_2_ hydrogenation to methane is a promising technology to store the excess of renewable energy into synthetic natural gas, benefiting from the existing natural gas transportation and storage infrastructure and well-established end-use technologies. Conventional processes operate at temperatures higher than 350 °C, with important drawbacks such as catalyst stability, increased CO production by the reversed water gas shift reaction (RWGS), and high energy consumption. The interest in operating at low temperatures (i.e., below 200 °C) to overcome these limitations has been considered by several authors^1−8^, and it is supported by an in-depth techno-economic simulation analysis performed by Yang et al.^1^ Nevertheless, a low-temperature methanation process requires overcoming the kinetic limitation of CO_2_ activation. Ru-based catalysts are, in general, more active than Ni ones; however, temperatures above 250 °C are still required to achieve competitive results.^9^ Recently, we reported a novel ruthenium oxycarbonate phase that activates CO_2_ at low temperatures (160–180 °C), with competitive methane production compared with state-of-the-art Ru and Ni catalysts.^10^ The active catalytic phase has been deeply characterized in our previous work at both macro and atomic scales and described as a carbon-doped RuO_2_, where C is located at interstitial positions of the ruthenium oxide monoclinic crystal phase, stabilizing low oxidation state Ru sites (Ru^*n*+^, 0 < *n* < 4). Removal of carbon species during the reaction leads to catalyst deactivation, setting a significant challenge for controlling the stabilization of C atoms in the oxycarbonate metastable phase with strong environment dependence. Tuning the catalytic performance in transition metal catalysts by the generation of interstitial atoms has already been reported in the literature,^11−14^ and its application in thermocatalysis can be found in examples such as in the selective hydrogenation of alkynes on Pd- and Ni-based catalysts,^11^ in methanol steam reforming on Cu based catalysts^15^ and in liquid phase selective hydrogenation and oxidation reactions.^16,17^ However, stabilizing interstitial atoms under demanding reaction conditions and controlling their generation at the atomic level remains challenging. In this work, based on operando spectroscopic studies, including advanced operando spectroscopic tools combined with kinetic studies, interstitial carbon atoms are successfully stabilized in the catalyst. Three sequential processes (carbon diffusion, metal oxide reduction, and decomposition of the oxycarbonate phase) are discussed, being influenced by the reaction conditions. This work represents a step forward in stabilizing the active ruthenium oxycarbonate phase, setting the key aspects for improving the stability of the catalyst.

## Results and Discussion

### Structural Catalyst Characterization

The catalyst labeled RuO_*x*_C_*y*_@C is prepared according to our very recent work.^10^ In brief, the catalyst is obtained by a hydrothermal synthesis method using RuO_2_, water, and glucose as reactants and submitted to 175 °C for 24 h. Spectroscopic results evidence the coexistence of three crystalline phases in the as-prepared material (ruthenium oxycarbonate (∼60%) labeled as RuO_2_C_*y*_, RuO_2_ (∼30%), and Ru^0^ (∼10%)). In addition, an amorphous carbon shell surrounding the whole particle is also observed. Details of the catalyst characterization can be found in ref (10). It has been demonstrated that interstitial C atoms in the ruthenium oxycarbonate phase stabilize Ru^*n*+^ sites in a low oxidation state and that the coexistence of RuO_2_C_*y*_ and Ru^0^ is crucial for catalytic activity, playing a key role in CO_2_ and H_2_ activation, respectively. The practical impact of the catalyst has already been reported in our previous work^10^ and displayed in Figure S1. However, specific issues remained undetermined, such as the optimal proportion of both phases, the reaction intermediate species, and the catalyst stability under highly reductive reaction conditions, which are investigated in this work using operando spectroscopic tools combined with catalytic studies.

### Catalyst Stability Under Reaction Conditions: Operando Spectroscopic Study

**Figure 1:**
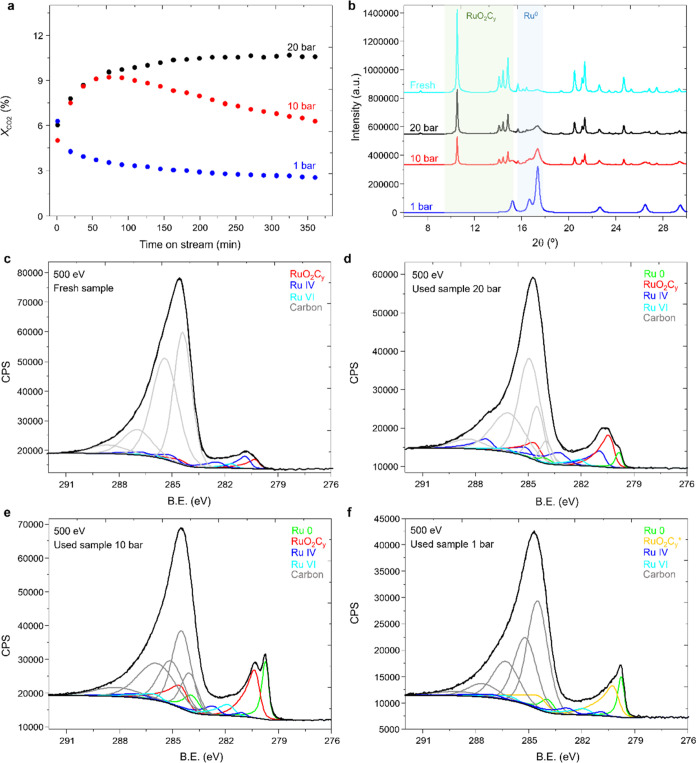
(a) Variation of the CO_2_ conversion with the time of stream on the RuO_*x*_C_*y*_@C catalyst at different pressures: 20 bar (black), 10 bar (red), and 1 (in blue) bar. Reaction conditions: 180 °C, H_2_:CO_2_ molar ratio 3:1 and GHSV 120 000 h^–1^. (b) Synchrotron XRD pattern of the RuO_*x*_C_*y*_@C catalyst in the fresh state (in cyan) and after exposure to 1 (in blue), 10 (in red), and 20 (in black) bars for ∼381 min of reaction. In color, the different zones include the characteristic peaks of RuO_2_C_*y*_ (green) and Ru^0^ (blue). For more information about the diffraction patterns of the different phases, see Figure S2 and Tables S1–S3. The evolution of the different phases with the reaction pressure is displayed in Figure S3. (c–f) Synchrotron XPS of the C 1s and Ru 3d core levels on fresh (c) and used samples after 20 bar (d), 10 bar (e), and 1 bar (f). X-ray excitation energy is 500 eV (i.e., depth 1.9 nm). Carbon in gray, RuO_2_C_*y*_ (in red), metallic ruthenium (labeled as Ru^0^, in green), oxidized ruthenium (labeled as Ru IV and Ru VI in dark blue and cyan, respectively), and a degraded RuO_2_C_*y*_ phase due to carbon or oxygen loss, labeled as RuO_2_C_y_* (in orange). The atomic fractions of each phase are graphically displayed in Figure S11.

This first section of the article presents a detailed analysis of the evolution under reaction conditions of both RuO_2_C_*y*_ and Ru^0^ phases to gain fundamental insights and learn how to control their amount by manipulating the reaction conditions while altering the corresponding catalytic efficiency. Thus, the impact of variables such as the reaction pressure, reactant feed composition, and contact time are investigated. The reaction takes place in a fixed-bed reactor (details in experimental section), and the reaction conditions used in our work are 180 °C, H_2_:CO_2_ molar ratio 3:1, and GHSV 120 000 h^–1^ for operating under differential conditions (i.e., conversion <10%). Under these conditions, the influence of the total reaction pressure (maintaining the molar composition of the feed constant) on the catalyst activity is studied and displayed in Figure 1a. An initial ∼6% CO_2_ conversion is observed in all cases. However, at 1 bar, the catalyst deactivates from an initial CO_2_ conversion of 6% to a final value of 2.6%. At 10 bar, the catalyst activity initially increases to 8.4% in the first 109 min of reaction but then decreases to a final value of 6.4%. At 20 bar, the activity increases in the initial 71 min of reaction up to 9.4% and remains constant during at least 361 min of the catalytic test.

Since it is clear that the catalyst evolves during the reaction conditions, we characterize the starting catalyst and the samples after having been exposed to 1, 10, and 20 bar for ∼361 min of reaction by Synchrotron X-ray Diffraction (SXRD), with the objective to follow the changes on the RuO_2_C_*y*_ and Ru^0^ phases during the reaction. Then, in Figure 1b cyan line, it can be seen that in the initial catalyst, the RuO_2_C_*y*_ phase dominates, while in the sample exposed to reaction at 1 bar (Figure 1b, blue line), only reflections of the metallic Ru^0^ are observed. In contrast, both RuO_2_C_*y*_ and Ru^0^ phases are visible for the samples exposed to the reaction at 10 and 20 bar (Figure 1b, red and black line, respectively). No separate RuO_2_ peaks are observed in the diffraction pattern due to peak overlapping and its low contribution (more details in ref (10)). Rietveld refinement of the SXRD pattern of the used sample at 20 bar shows an increased average amount of interstitial carbon atoms in the ruthenium oxycarbonate (RuO_2_C_*y*_) phase (approximate composition RuO_2_C_0.79±0.03_), compared to the sample reacted at 10 bar (RuO_2_C_0.51±0.03_) and the fresh sample (RuO_2_C_0.41±0.03_). In addition, the reaction pressure affects the amount of Ru metal, increasing from ∼10 wt % in the as-prepared fresh sample to ∼34, 55, and 100 wt % after the reaction at 20, 10, and 1 bar, respectively (Table S4). In parallel, an increase in their average crystal size, from 4 nm in the fresh sample to 6, 8, and 13 nm after reaction at 20, 10, and 1 bar, respectively, is observed. In definitive, from the SXRD results, it can be deduced that carbon diffusion is promoted at increasing reaction pressure, hindering the reduction of oxidized ruthenium species to ruthenium metal.

**Figure 2:**
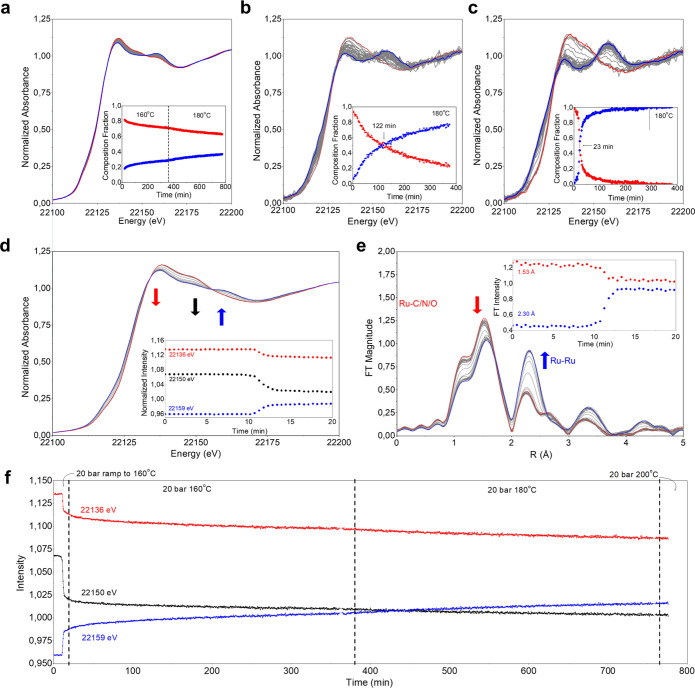
(a–c) Ru K-edge XANES temporal evolution of the RuO_*x*_C_*y*_@C system under operando CO_2_ + H_2_ conditions at 160/180 °C for 770 min at 20 bar (a), 180 °C for 370 min at 10 bar (b), and 180 °C for 370 min at 1 bar (c). The insets report the evolution of fractional contribution obtained by MCR-ALS analysis (details in the SI) over time, where in red and blue represent the RuO_2_C_*y*_ and Ru^0^ phases, respectively. (d) Ru K-edge XANES spectra of catalyst at 20 bar under reaction conditions (CO_2_ + H_2_) ramping to 160 °C. Inset: time evolution of the signal at 22 136, 22 150, and 22 159 eV chosen to maximize the spectral differences between RuO_2_C_*y*_, RuO_2_, and metallic Ru^0^. (e) Fourier transformed *k*^2^ weighted EXAFS spectrum in a *k*-range of 3–15 Å^–1^ with a Hannings window showing the catalyst at 20 bar ramping under reaction conditions to 160 °C. (f) Intensity traces at 22136 eV (red), 22150 eV (black), and 22159 eV (blue) best correlate to RuO_2_C_*y*_, RuO_2_, and Ru^0^, respectively.

The evolution of ruthenium species under reaction conditions is followed by operando Ru K-edge X-ray absorption spectroscopy (XAS) performed at 1, 10, and 20 bar. In agreement with the ex situ SXRD data collected on the used samples, a progressive transformation of the starting RuO_2_C_*y*_ phase to Ru^0^ is observed (Figure 2a–c). The ex situ Ru K-edge X-ray absorption near-edge structure (XANES) spectra collected on the RuO_*x*_C_*y*_@C catalyst and the used material at 1, 10, and 20 bar are reported in the Supporting Information compared with Ru^0^ and RuO_2_ references (Figures S6 and S7) and match with the operando data reported in Figure 2a–c at different working pressures. The insets of Figure 2a–c report the evolution of the RuO_2_C_*y*_ (red) and Ru^0^ (blue) fractional contributions under reaction conditions obtained by multivariate curve resolution alternating least-squares (MCR-ALS) analysis (more details in the Supporting Information and Figure S6) as a function of time. Notice that only two components are needed to describe the changes observed (Figure 2a–c) due to the overlapping features of RuO_2_ and RuO_2_C_*y*_. The progressive transition from the starting ruthenium species to Ru^0^ is promoted at 1 bar, delayed, and slightly inhibited at 10 bar, while strongly inhibited at 20 bar. In particular, the MCR-ALS analysis shows that at 1 and 10 bar after 360 min at 180 °C, ∼100 and ∼70% of RuO_2_C_*y*_ are converted to Ru^0^, respectively. In contrast, at 20 bar, only ∼30% is converted after 360 min at 160 °C, reaching 36% after an additional 360 min at 180 °C, in line with SXRD data. Moreover, not only is the total amount of Ru^0^ formed at lower pressure higher, but the rate of formation is also faster, being significantly faster at 1 bar than 10 bar, which in turn is faster than at 20 bar (more kinetic details in the SI). Indeed, MCR-ALS analysis of the operando data shows a crossover point at 23 min for the reaction at 1 bar, while at 10 bar, this is observed much later at 122 min, with the progression to Ru^0^ being much slower and much more delayed at 20 bar.

Globally, the reported results confirm the stabilization of RuO_2_C_*y*_ and the inhibition of ruthenium reduction at an increasing reaction pressure.

In Figure 2d–f, a detailed analysis of the operando XAS data at 20 bar is presented, displaying the progressive transformation of ruthenium species under reaction conditions at an increasing temperature from 160 to 200 °C. To help deconvolute the contributions from RuO_2_ and RuO_2_C_*y*_, which have overlapping features, PCA and MCR-ALS are performed, revealing a dominant conversion of RuO_2_ to Ru^0^ at the initial step. Metallic Ru^0^ formation upon ramping up the temperature to 160 °C over 20 min is evidenced by the intensity increases at 22 159 eV (due to Ru^0^, Figure 2f blue line) in the XANES region and a corresponding increase at 2.3 Å in the *r*-space of the FT spectra due to Ru–Ru scattering. Simultaneously, a slight decrease at 22 136 eV and a more pronounced one at 22 150 eV are observed in the XANES, which correlate to RuO_2_C_*y*_ (Figure 2f red line) and RuO_2_ (Figure 2f black line), respectively, as well as a decrease at 1.7 Å in the FT spectra due to Ru–C/O scattering. After the initial temperature ramp of 12 min, where such transformations occur, further changes in the material are minimal over the duration of the experiment, ∼10 h, even after further temperature increases from 160 to 180 °C and then to 200 °C (Figure 2f and more details in the SI). Furthermore, as described above, these changes can also be described by a two-component system. These results are in line with ex situ SXRD results showing that RuO_2_C*_y_* and Ru^0^ coexist and stabilize in the sample used at 20 bar.

Finally, to find out the role of the reactant feed in the overall process, a similar experiment at 20 bar replacing CO_2_ and H_2_ by He is performed (Figure S7). When this is done, changes in the catalyst started to occur at ∼160 °C, and the formation of Ru^0^ is evidenced both from the rise in intensity at 2.3 Å (FT) and at 22159 eV (XANES). Interestingly, in the first ∼50 to 60 min of the reaction under He flow, it seems that more RuO_2_ forms together with Ru^0^ at the expense of the RuO_2_C_*y*_ phase, while once it reached 200 °C (at time ∼200 min), the Ru^0^ forms at the expense of both RuO_2_ and the RuO_2_C_*y*_ phases. These results reveal that the CO_2_ reactant feed plays a crucial role in the stability of the RuO_2_C_*y*_ phase when operating at 20 bar (see the SI).

Complementary to bulk techniques such as SXRD and XAS data, surface sensitivity is obtained using synchrotron X-ray photoelectron spectroscopy (XPS) analysis at different sampling depths (1.9 and 5.6 nm) (details in the SI). The coexistence of ruthenium oxycarbonate, RuO_2_, and Ru^0^ phases are observed on the upper layers of the catalyst surface on the fresh and used samples (Figure 1c–f), with the percent of Ru^0^ being higher in the samples after 1 and 10 bar (Table S6 and Figures S10 and S11). Nevertheless, after 1 bar of reaction, a shift of the BE ascribed to RuO_2_C_*y*_ from 280.3 eV to lower values (i.e., 280.1 eV) is observed, which may correspond to a partial structure degradation due to C or O removal. Supporting this assumption, operando Infrared studies performed at 1 bar in the far-infrared region (FIR) using synchrotron radiation on the AILES beamline of the SOLEIL synchrotron light source show a gradual decrease with reaction time of the IR bands at 278 and 375 cm^–1^ associated with the ν(Ru–O–C) vibration of the Ru-CO_3_ vibrational group^18−20^ (Figure S12). The reduction of these two bands can be linked to partial structure degradation due to the elimination of interstitial carbon. The gradual decrease of the Ru-CO_3_ bands in about 10 min agrees with the decrease of XANES RuO_2_C_*y*_ at similar temperature and pressure conditions.

**Figure 3:**
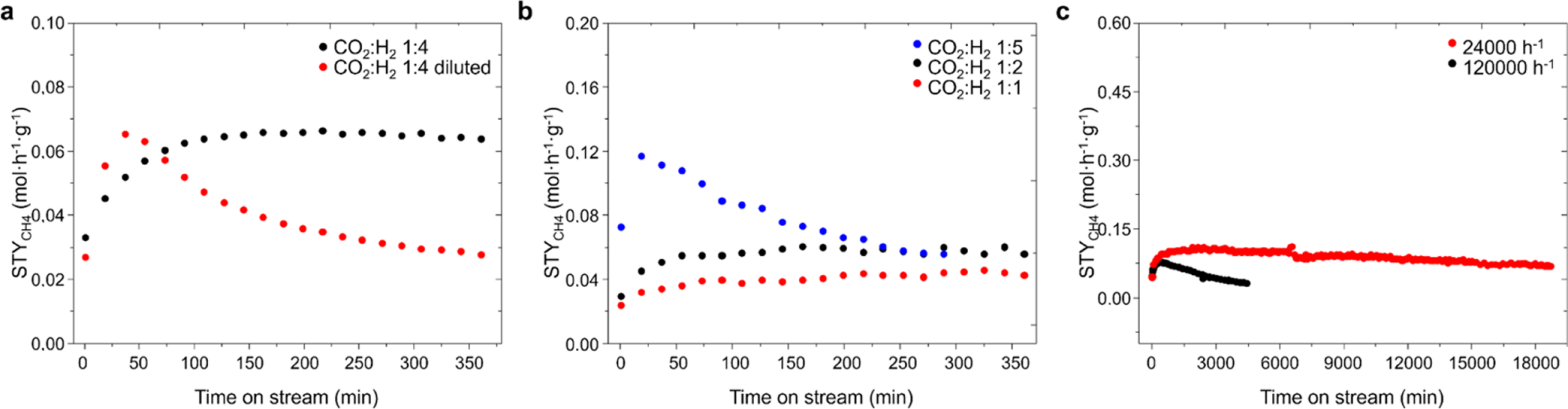
Variation of the yield to methane with the time on stream on the RuO_*x*_C_*y*_@C catalyst at 180 °C and 20 bar under different reaction conditions. (a) Influence of the partial pressure of CO_2_ at a constant 1:4 CO_2_:H_2_ molar ratio. Black line: CO_2_:H_2_ = 1:4 (19 vol % CO_2_, 76 vol % H_2_, 5 vol % N_2_), and red line: CO_2_:H_2_ = 1:4 diluted (5 vol % CO_2_, 20 vol % H_2_, 75 vol % N_2_). GHSV 120 000 h^–1^. (b) Influence of the partial pressure of CO_2_ at a constant partial pressure of H_2_ resulting in different CO_2_:H_2_ molar ratios. Blue line, CO_2_:H_2_ = 1:5 (9.5% CO_2_, 47.5% H_2_, 43% N_2_), black line, CO_2_:H_2_ = 1:2 (23.7% CO_2_, 47.5% H_2_, 28.8% N_2_), and red line, CO_2_:H_2_ = 1:1 (47.5% CO_2_, 47.5% H_2_, 5% N_2_). GHSV 120 000 h^–1^. (c) Influence of the gas hour space velocity (GHSV). Red line: 24 000 h^–1^ (CO_2_:H_2_ = 1:3) and black line: 120 000 h^–1^ (CO_2_:H_2_ = 1:3).

Based on the multitechnique results reported above, it is clear that the ruthenium oxycarbonate phase is stabilized under reaction conditions at 20 bar, while at lower pressure, Ru^0^ predominates. Ru^0^ alone has been shown to be inactive under our reaction conditions,^10^ explaining the loss of activity when Ru^0^ is formed. As mentioned in our previous work,^10^ the reduced catalyst can be easily regenerated by submitting it to an oxidizing treatment under controlled conditions. Next, we realized that the stabilization of the ruthenium oxycarbonate phase is related to the partial pressure of CO_2_ and H_2_. This is demonstrated in Figure 3a, where at a total pressure of 20 bar, decreasing the CO_2_ partial pressure from 3.8 bar (i.e., 19 vol % CO_2_) to 1 bar (i.e., 5 vol % CO_2_) and maintaining the CO_2_:H_2_ ratio equal to 1:4, the catalyst deactivates with time on stream. Additional experiments are done studying the effect of the partial pressure of each reactant separately while keeping the other unaltered at a constant pressure of 20 bar (see the SI). As shown in Figure S15a,c at constant *P*_CO_2__ = 4.7 bar (i.e., 23.7 vol % CO_2_), the apparent order of H_2_ is close to zero, which explains that the methane reaction rate is independent of the H_2_ partial pressure, at least in the range between 4.7–15.2 bar (i.e., 23.7–76 vol % H_2_). However, when varying the CO_2_ partial pressure at a constant *P*_H_2__ = 9.5 bar (i.e., 47.5 vol % H_2_) (Figure S15b,d), an apparent order of −0.4 is observed, which translates as a negative contribution to the rate of methane formation. This can be explained from a kinetic point of view as an effect of the surface coverage of carbon-based intermediates, reducing the availability of active H on the surface (i.e., competitive adsorption). Furthermore, from a chemical/structural point of view, a higher CO_2_ partial pressure is convenient for the stability of the catalyst (Figure 3b), while at low CO_2_ partial pressure (*P*_CO_2__ = 1.9 bar) (i.e., 9.5 vol % CO_2_) and a high H_2_:CO_2_ ratio (i.e., 5:1), the catalyst deactivates (Figure 3b, blue line). This deactivation process is related to a reduction of the initial ruthenium phases to Ru^0^, as demonstrated by XRD (Figure S16b). Therefore, it is important to achieve a high enough coverage of carbon-based intermediates with a not-too-high H_2_ coverage so that structural or chemical changes in the catalyst may not appear.

Another critical parameter to consider is the space velocity of the reactants in contact with the catalyst. As shown in Figure 3c, higher catalyst stability is observed when operating at a low space velocity, maintaining the rest of the variables constant. Similar experiments operating at a lower space velocity (i.e., 24 000 h^–1^ instead of 120 000 h^–1^) are done at 1 and 10 bar, trying to retain catalyst stability. As shown in Figure S13, the deactivation rate is depressed at 10 bar but still not at 1 bar.

In summary, considering the above spectroscopic and catalytic results, three processes can occur in the operating catalyst, and the relative rates are influenced by the reaction conditions: (i) carbon diffusion into the metal oxide and/or oxycarbonate lattice, i.e., RuO_2_ + *x*C = RuO_2–3*x*_(CO_3_)_*x*_; (ii) reduction of the metal oxide and/or oxycarbonate phase to Ru^0^, i.e., RuO_2_ + H_2_ = Ru^0^ + H_2_O and RuO_2–3*x*_(CO_3_)_*x*_ + H_2_ = Ru^0^ + *x*CO_2_ + H_2_O; and (iii) metal oxycarbonate phase decomposition, i.e., RuO_2–3*x*_(CO_3_)_*x*_ = (1 – *x*) RuO_2_ + *x*Ru^0^ + *x*CO_2_. At high CO_2_ pressure and low space velocity, the first process is promoted, and the last one is suppressed, resulting in a stable methane production with reaction time (See Figures 1a and 3a,c). In contrast, the second process is favored if the H_2_ concentration is too high and the concentration of CO_2_ is not high enough to compensate for the global process through reaction (i), leading to catalyst deactivation (see Figures 1a and 3b). Moreover, in the absence of reactants, for instance, in Helium flow, the last process takes place. These results show the possibility of stabilizing the active RuO_2_C_*y*_ phase, hindering further reduction to Ru^0^ by controlling the reaction conditions.

C diffusion and incorporation in the metal lattice can be understood if CO is formed as an intermediate species and is further dissociated into C and O atoms. However, this is difficult to discern from the catalytic data since gas chromatogram (GC) analysis does not detect CO during gas-phase reaction conditions. Thus, transient kinetic experiments have been carried out to gain a deeper understanding of the reaction mechanism, setting the working pressure to 10 bar and the temperature to 140 °C. As shown in Figure S19, after switching from a CO_2_/N_2_ feed to an H_2_/N_2_ reactant feed, CO is immediately detected by mass spectrometry. Subsequently, H_2_O and CH_4_ are formed at rates lower than those of CO (details in the SI). These results confirm that CO is formed on the surface and can be an intermediate in CH_4_ formation. In this transient study, some of the CO is desorbed from the catalyst surface, probably due to competitive adsorption of H_2_, while another CO fraction is further hydrogenated to CH_4_. In order to achieve a better understanding of the role that CO plays in the reaction mechanism, additional experiments are done where CO is cofed along with the reaction mixture.

### Addition of CO in the Reactant Feed

**Figure 4:**
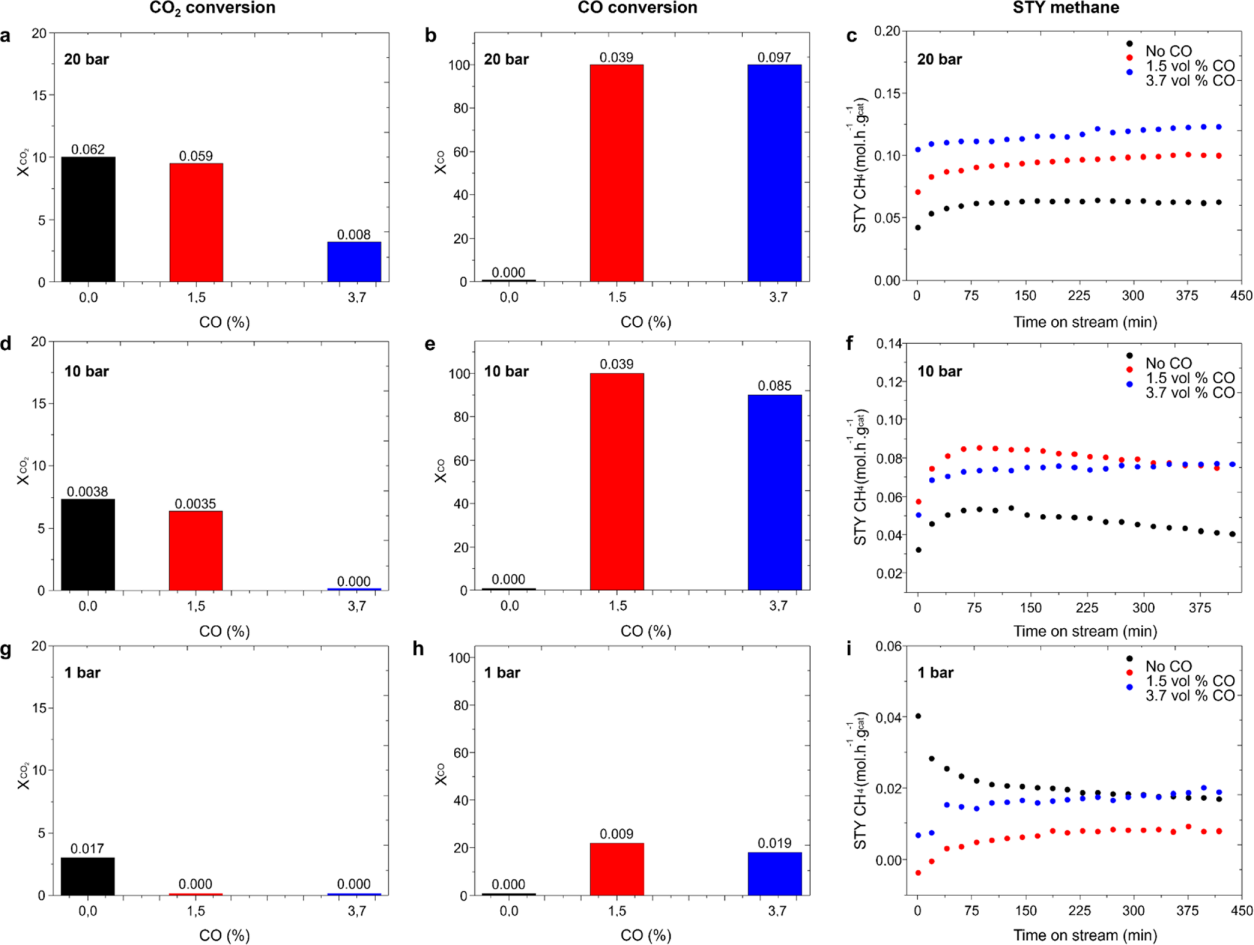
(Left panel) Separate CO_2_ conversion at different CO percent (1.5 and 3.7% vol) in the reactant feed and reaction pressures: 20 (a), 10 (d), and 1 (g) bar. (Middle panel) Separate CO conversion at different CO percent (1.5 and 3.7% vol) in the reactant feed and reaction pressures: 20 (b), 10 (e), and 1 (h) bar. (Right panel) Methane production (mol/h·g_cat_) in the presence of different amounts of CO in the reactant feed (1.5% CO in red, and 3.7% CO in blue) and at different reaction pressures: 20 (c), 10 (f), and 1 (i) bar. Reaction conditions: 180 °C, GHSV 120 000 h^–1^, and CO_2_:H_2_ 1:3. The numbers above the bars indicate the associated methane production in mol·h^–1^·g^–1^ based on each CO_2_ and CO conversion.

The role of CO has been widely discussed in the literature, and it can be considered a poison in many processes due to a strong interaction with the active site,^21,22^ or it can behave as a promoter by removing water through the water–gas shift reaction or by keeping the catalyst in a reduced state.^23^ In our case, a positive effect is observed on the activity but, most interestingly, on the catalyst stability. In this direction, experiments at 1, 10, and 20 bars are done by coadding CO in the reactant feed (1.5 and 3.7 vol %) (details in the SI). At both 20 and 10 bar, added CO is rapidly and practically totally converted (Figure 4), resulting in enhanced methane production. However, at 1 bar reaction pressure, the CO introduced is converted in a lower extension (with ∼22 to 18% CO conversion), with a lower effect on methane production. In addition, as clearly illustrated in Figure 4, a competitive CO versus CO_2_ hydrogenation is observed, the extension of which depends on the operating pressure. Thus, at 1 bar, CO competes over CO_2_, resulting in 0% CO_2_ conversion, whereas this effect is less pronounced at 20 bar. Similar behavior has already been observed in the literature and explained by a site-blocking effect of the more strongly adsorbed CO, preventing CO_2_ adsorption.^21^ Notice also that adding CO results in a slight decrease of the selectivity to CH_4_ (down to 97 at 3.7% CO and 1 bar), with the formation of other subproducts such as C_2_–C_5_ hydrocarbons, isopropanol, and acetone, specifically at the higher CO % (details in the SI, Figure S20).

**Figure 5:**
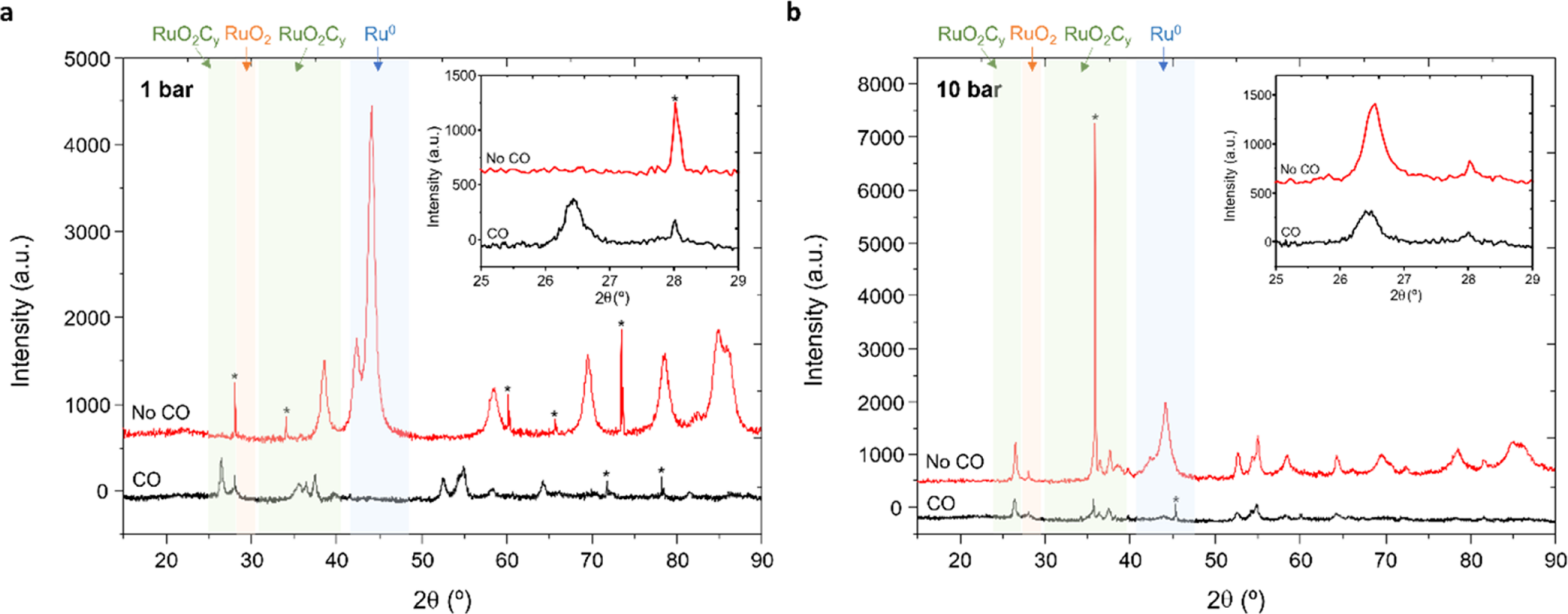
XRD pattern of the samples after being exposed to 1 (a) and 10 (b) bars without CO (in red) and by coadding 3.7% CO (in black). The insets are the expansion of the XRD patterns centered at the ∼26° peak characteristic of the RuO_2_C_*y*_ phase. The asterisk indicates peaks due to the SiC used in the catalytic studies. In color, the different zones correspond to the characteristic peaks of RuO_2_C_*y*_ (green), RuO_2_ (red), and Ru^0^ (blue). For more information about the diffraction patterns of the different phases, see Figure S2 and Tables S1–S3.

However, an important aspect is that the cofeeding of CO positively affects the catalyst stability. This is clearly shown in Figure 4f,i, where at 10 and 1 bar, the catalyst tends to deactivate in the absence of CO (black line), whereas the catalyst stabilizes in the presence of CO (red and blue line). This is due to RuO_2_C_*y*_ stabilization as reflected in the XRD pattern of the used samples (Figure 5). Hence, Ru^0^ is predominately observed in the sample after reaction at 1 and 10 bar in the absence of CO, whereas in the presence of CO, RuO_2_C_*y*_ is stabilized, probably due to the dissociation of CO into C and O, which is proposed to take place on the ruthenium metal component of the catalyst. The surface carbon atoms generated under reaction conditions act as a reservoir that enables replenishing interstitial generated vacancies in the RuO_2_ crystal lattice with carbon atoms, stabilizing the oxycarbonate active phase under steady-state conditions and contributing to retaining catalyst activity over long-term operation. In this way, the reductive degradation of the active oxycarbonate phase is suppressed.

### Influence of the Catalyst Composition

It has been indicated that the coexistence of RuO_2_C_*y*_ and Ru^0^ is crucial for catalytic activity, playing a key role in CO_2_ and H_2_ (and CO) activation, respectively. The impact that the ratio of the two phases has on the catalytic performance is examined in this section of the work in order to determine and achieve an optimum.

**Figure 6:**
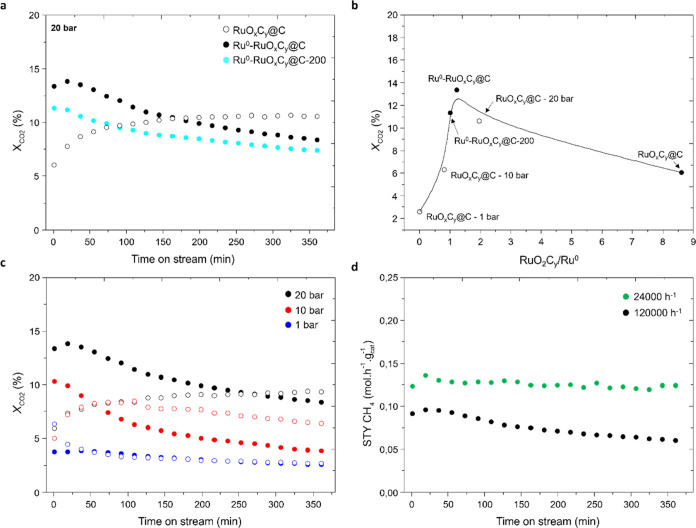
(a) Comparative study of the variation of the CO_2_ conversion at 20 bar with time on stream on RuO_*x*_C_*y*_@C (empty black circles), Ru^0^-RuO_*x*_C_*y*_@C (full black circles), and Ru^0^-RuO_*x*_C_*y*_@C-200 (full cyan circles) samples, at GHSV 120 000 h^–1^, 180 °C, and CO_2_:H_2_ 1:3. (b) Relative amount of RuO_2_C_*y*_/Ru^0^ determined from the XRD pattern of the fresh (RuO_*x*_C_*y*_@C, Ru^0^-RuO_*x*_C_*y*_@, and Ru^0^-RuO_*x*_C_*y*_@C-200, full circles) and the used RuO_*x*_C_*y*_@C samples (after 20, 10, and 1 bar, empty circles) versus the initial catalyst activity for methane production. (c) Comparative study of the influence of the pressure, 20 (in black), 10 (in red), and 1 (in blue) bar, on the CO_2_ conversion with the time on stream on Ru^0^-RuO_*x*_C_*y*_@C (full circles) compared to the RuO_*x*_C_*y*_@C (empty circles) sample, at GHSV 120 000 h^–1^, 180 °C, and CO_2_:H_2_ 1:3. (d) Influence of the gas velocity on the yield to methane with time on stream on the Ru^0^-RuO_*x*_C_*y*_@C sample. Black line, GHSV of 120 000 h^–1^, and green line, GHSV of 24 000 h^–1^. Reaction conditions: 20 bar, 180 °C, CO_2_:H_2_ 1:3.

For this purpose, we synthesize two additional catalysts labeled Ru^0^-RuO_*x*_C_*y*_@C and Ru^0^-RuO_*x*_C_*y*_@C-200 with a higher fraction of Ru^0^ versus RuO_2_ and RuO_2_C_*y*_ in the starting precursor (see Table S6). In both cases, a RuO_2_ precursor with a slightly lower particle size (24 nm vs. 39 nm) is employed in order to promote the partial reduction of RuO_2_ to Ru^0^ under the hydrothermal synthesis conditions (see the TPR-H_2_ pattern in Figure S21 and details in SI). The synthesis temperature is raised to 200 °C in the Ru^0^-RuO_*x*_C_*y*_@C-200 compared to the previous 175 °C to promote the fraction of Ru metal (more details in SI). As shown in Figure 6a, the newly prepared samples have a higher initial CO_2_ conversion at 20 bar than the reference RuO_*x*_C_*y*_@C sample, and when comparing the initial activity of the fresh catalysts with the ratio of crystalline phases determined from the XRD patterns, a tentative Gaussian line shape is observed, indicating that an adequate balance between Ru^0^ and RuO_2_C_*y*_ promote catalyst activity (Figure 6b, full circles). It is also interesting to notice that the RuO_*x*_C_*y*_@C used catalysts after 1, 10, and 20 bar (Figure 6b, empty circles) perfectly match the same Gaussian curve.

In contraposition to the higher initial activity, both Ru^0^-RuO_*x*_C_*y*_@C and Ru^0^-RuO_*x*_C_*y*_@C-200 catalysts suffer a substantial deactivation with time on stream at 20, 10, and 1 bar (Figure 6a,c). In order to rationalize this deactivation pattern, an in-depth characterization study is done on the Ru^0^-RuO_*x*_C_*y*_@C catalyst. XRD (Figure S22) and depth profile XPS analysis (Figure S23 and Table S5) done on the fresh and used samples demonstrate that the fraction of Ru^0^ has further increased after the reaction. In detail, the amount of surface Ru^0^ determined from XPS at a sampling depth of 1.9 nm is 3.8% in the Ru^0^-RuO_*x*_C_*y*_@C sample after reaction at 20 bar, in strong contrast with the 0.6% observed in the 20 bar RuO_*x*_C_*y*_@C sample (more details in the SI). From these results, we speculate that the presence of a high amount of Ru metal in the catalyst composition promotes H_2_ activation (see H_2_/D_2_ exchange experiments in Table S8), resulting in high H surface coverage under working conditions, hampering the stability of the RuO_2_C_*y*_ phase. In fact, operando XAS studies at 1 and 10 bar (Figure S25) show a much faster transition from the starting ruthenium species to Ru^0^ in the Ru^0^-RuO_*x*_C_*y*_@C sample compared to the previous RuO_*x*_C_*y*_@C. Quantitative conversion of the starting ruthenium species to Ru^0^ in the Ru^0^-RuO_*x*_C_*y*_@C sample is observed at 40 min for 1 bar and 50 min at 10 bar in opposite to the ∼145 min and >400 min, respectively for the RuO_*x*_C_*y*_@C sample (Figures S26 and S27). Returning to the catalytic data shown in Figure 6c, where the catalysts deactivate during the progress of the reaction, we demonstrate the possibility of overcoming the limited catalyst stability by decreasing the space velocity (Figure 6d, green line). This result has significant consequences, specifically regarding catalyst stability, which is crucial for long-term applications. Based on it, an optimum RuO_2_C_*y*_/Ru^0^ ratio of 1.2 wt % (determined from the XRD pattern of the fresh material) is observed, enabling high activity and stability under controlled reaction conditions.

## Conclusions

In summary, in this work, catalyst optimization considering critical aspects such as the stabilization of interstitial carbon species in the ruthenium oxycarbonate (i.e., RuO_2_C_*y*_) phase is studied by combining operando spectroscopic studies with catalytic and kinetic studies. It is proposed that the surface coverage of carbon intermediate species under reaction conditions, which is influenced by the CO_2_ partial pressure, is a critical parameter for stabilizing the oxycarbonate active phase under steady-state conditions, contributing to retaining catalyst activity over long-term operation. Following the above, we demonstrate that adding small amounts of CO in the reactant feed positively affects the stability of the oxycarbonate phase, increasing both the catalyst activity and stability. This has important application in its practical application, with the possibility of operating at lower pressure (1 bar) eliminating the need to run at high pressure in order to increase stability. Based on these outcomes, it is possible to control the stability of surface ruthenium oxycarbonate species and its catalytic performance and evolution with time on stream by an adequate selection of the reaction conditions and catalyst composition. In addition, we define an optimum in the catalyst composition between the RuO_2_C_*y*_ and Ru^0^ phases, a low amount of Ru metal being beneficial, whereas a high fraction of Ru^0^ in the catalyst composition promotes H_2_ activation, resulting in a high H surface coverage under working conditions, hampering the stability of the RuO_2_C_*y*_ phase. This study also indicates that CO is an intermediate species with a significant surface coverage. A fraction of the CO is proposed to dissociate on the Ru metal fraction into C and O atoms, where these C atoms act in part as a reservoir stabilizing the ruthenium oxycarbonate phase. Combining all of the results, it can be explained how the catalyst composition and reaction conditions play a critical role in determining the activity and stability of the catalyst.

The information gathered from this work constitutes the basis for designing a more active and stable catalyst with the possibility to operate at lower pressure and low temperature. In particular, we describe the possibility of improving the catalyst stability at 1 bar by adding CO to the reactant feed.

## Methods

### Synthesis

The RuO_*x*_C_*y*_@C sample is prepared using the synthetic procedure described in ref (10). Briefly, the catalyst is synthesized using 120 mg of glucose (Aldrich, >99.5%), 7 mL of miliQ water, and 100 mg of RuO_2_ (Aldrich, 39 nm). Then, all of the chemicals are loaded into a Teflon-coated stainless-steel autoclave of 15 mL and placed in an oven at 175 °C under static conditions for 24 h. Afterward, the autoclave is cooled to room temperature for 2 h. The black solid of the autoclave is filtrated and washed with abundant distilled water and acetone. It is then dried in an oven at 60 °C overnight. Ru^0^-RuO_*x*_C_*y*_@C is prepared as RuO_*x*_C_*y*_@C but with a different RuO_2_ precursor (Alfa Aesar, 24 nm). Ru^0^-RuO_*x*_C_*y*_@C-200 is prepared as RuO_*x*_C_*y*_@C but at a higher oven temperature (i.e., 200 °C).

### Catalyst Characterization

X-ray powder diffraction is recorded with a Philips X́Pert diffractometer using monochromatic Cu Kα radiation (λ = 0.15406 nm).

Synchrotron XPS experiments are performed on beamline BL-24 (CIRCE) at the ALBA Synchrotron Light Facility. The end station is equipped with a Phoibos 150 NAP electron energy analyzer (SPECS GmbH). The beam size is 100 × 30 (height × volume) μm^2^, with a pass energy of 10 eV, a step of 0.1 eV, and a beamline exit slit of 20 μm. Synchrotron XPS studies are performed in order to obtain depth profile analysis. Incident photon energies of 500 and 1.400 eV for Ru 3d and C 1s are used, resulting in a sample probing depth of 1.9 and 5.6 nm, respectively. More details of sample preparation and spectra acquisition are in the Supporting Information.

Synchrotron XAS experiments are performed with the BL-22 beamline (CLAESS) at the ALBA Synchrotron Light Facility as well as at the Advanced Photon Source (APS) 10- BM-A, B beamline of the Argonne National Laboratory for data at 20 bar. The samples are diluted in boron nitride and then pelletized. The spectra are collected in transmission mode using a homemade catalytic cell, allowing us to record XAS spectra under reaction conditions (1–20 bar) and (25–200 °C) and connected online to a mass spectrometer (OMNIStar GSD 320 Gas Analysis System from Pfeiffer). For further information, see the Supporting Information. XAS spectra were collected in transmission mode with the Si311 double-crystal monochromator available at the beamline. The spectra were acquired at 80 K up to a high *k* value (20 Å^–1^) to access a higher sensitivity to the local structure. Several XAS repeats were collected to ensure reproducibility and statistics, and the averaged spectra were treated with the Athena software package. The energy scale was calibrated by setting the first inflection point of the Ru black spectra at 22117 eV. The EXAFS oscillations were extracted using the AUTOBK algorithm employing a spline in the 0–19 Å^–1^ region of the *k* space with an *R*_bkg_ of 1. The FEFF6 code is used for the scattering path generation, and multi- (*k*^1^, *k*^2^, and *k*^3^)-weighted fits of the data were carried out in *r*-space over the *r* and *k* ranges, as indicated in Supplementary Table S5. The *S*_0_^2^ value was set at 0.9, and *E*_0_ was refined with the initial *E*_0_ value set to the first inflection point of the rising edge. Scattering paths were fit in terms of Δ*R*_eff_ and σ^2^, which represent deviations from the expected interatomic distances and the structural disorder, respectively. To assess the goodness of the fits, both the *R* factor (% *R*) and the reduced χ^2^ were minimized, which ensured that the data were not overfitted. Best-fit models were determined by using a grid search with fixed values for the path coordination numbers (*N*) by employing Larch, the Python implementation of Artemis. The detailed description of the EXAFS modeling is reported in the Supporting Information.

SXRD data are collected at the BL-04 MSPD beamline at the ALBA Synchrotron Light Facility. The measurements are carried out using a MYTHEN position-sensitive detector at 20 keV photon energy (wavelength 0.6201 Å refined using the NIST 640d standard).

Synchrotron IR spectra were performed at the AILES Beamline of SOLEIL Synchrotron Light Facility (Saint Aubin, Paris, France). The IR spectra were acquired by using a Bruker IFS 125 MR spectrometer. The experiment was carried out by using a homemade cell, allowing the recording of infrared reflection–absorption spectra under various catalytic conditions of pressure (up to 30 bar) and/or temperature (up to 900 °C). A detailed description of the FIR setup is reported in the Supporting Information.

### Catalytic Studies

CO_2_ was performed in a stainless-steel fixed-bed reactor with an inner diameter of 11 mm and a length of 240 mm. Typically, 90 mg of catalyst (particle size 400–600 μm) is diluted in SiC at a weight ratio of 0.04 (catalyst/SiC) and mounted inside the reactor without prior activation. The reaction takes place at variable pressure (i.e., 20, 10, or 1 bar), 180 ± 5 °C, and GHSV of 120 000 h^–1^. Additional experiments are done at higher catalyst loading, resulting in a GHSV of 24 000 h^–1^. The feed is 23.8 vol % CO_2_, 71.3 vol % H_2_, and 5 vol % N_2_ (1:3 CO_2_:H_2_). Direct analysis of the reaction products is done by online gas chromatography using SCION-456-GC equipped with thermal conductivity (MS-13X column) and flame ionization (BR-Q Plot column) detectors. Blank experiments (in the presence of SiC) show the absence of a homogeneous contribution to the reaction. The transient studies were carried out at 10 bar, 140 °C, and GHSV of 16 500 h^–1^, and the reaction products were analyzed online by mass spectrometry (MS, Omnistar GSD 320). A detailed description of each catalytic experiment is reported in the Supporting Information.

## Data Availability

All data is available in the manuscript or the Supporting Information.

## Supporting Information Available

Synthesis of RuO_*x*_C_*y*_@C, spectroscopic characterization including X-ray diffraction (XRD), synchrotron X-ray diffraction (SXRD), X-ray adsorption spectra (XAS), synchrotron X-ray photoelectron spectroscopy (XPS), synchrotron far-infrared studies (FIR) of fresh and used RuO_*x*_C_*y*_@C catalysts at 1, 10, and 20 bar, catalytic performance of the RuO_*x*_C_*y*_@C catalyst including stabilization of the RuO_2_C_*y*_ phase with the partial pressure of CO_2_, analysis of initial reaction rates, influence of partial pressure of H_2_ and CO_2_ on the reaction rate and catalyst stability, space velocity and its influence on the catalyst performance, CO_2_ hydrogenation transient study and CO_2_ hydrogenation in the presence of CO, Ru^0^-RuO_*x*_C_*y*_@C catalyst including synthesis, precursors’ TPR-H_2_, and spectroscopic characterization including X-ray diffraction (XRD), X-ray adsorption (XAS), and synchrotron X-ray photoelectron spectroscopy (XPS) of fresh and used samples at 1, 10, and 20 bar, Ru^0^- RuO_*x*_C_*y*_@C-200 catalyst including synthesis and spectroscopic characterization using XRD, and hydrogen/deuterium (H_2_/D_2_) exchange experiments (PDF)

## References

[ref1] Lee W. J.; Yang Y.; et al. Recent trend in thermal catalytic low temperature CO_2_ methanation: A critical review. Catal. Today 2021, 368, 2–19. 10.1016/j.cattod.2020.02.017.

[ref2] Inoue M.; et al. Structure-Sensitivity Factors Based on Highly Active CO_2_ Methanation Catalysts Prepared via the Polygonal Barrel-Sputtering Method. J. Phys. Chem. C 2020, 124, 10016 10.1021/acs.jpcc.0c01666.

[ref3] Chen S.; et al. Electronic metal-support interactions and their promotional effect on CO_2_ methanation on Ru/ZrO2 catalysts. J. Catal. 2021, 400, 407 10.1016/j.jcat.2021.06.028.

[ref4] Cored J.; et al. Hydrothermal Synthesis of Ruthenium Nanoparticles with a Metallic Core and a Ruthenium Carbide Shell for Lowerature Activation of CO_2_ to Methane. J. Am. Chem. Soc. 2019, 141, 19304 10.1021/jacs.9b07088. 31774282

[ref5] Polanskia J.; et al. Oxide passivated Ni-supported Ru nanoparticles in silica: A new catalyst for low-temperature carbon dioxide methanation. Appl. Catal., B 2017, 206, 16–23. 10.1016/j.apcatb.2017.01.017.

[ref6] Siudyga T.; et al. Ultra-low temperature carbon (di)oxide hydrogenation catalyzed by hybrid ruthenium-nickel nanocatalysts: Towards sustainable methane production. Green Chem. 2020, 22, 5143 10.1039/D0GC01332C.

[ref7] Yamada K.; Ogo S.; Yamano R.; Higo T.; Sekine Y. Low-temperature Conversion of Carbon Dioxide to Methane in an Electric Field. Chem. Lett. 2020, 49, 303 10.1246/cl.190930.

[ref8] Fukuhara C.; et al. Auto-methanation for transition-metal catalysts loaded on various oxide supports: A novel route for CO_2_ transformation at room-temperature and atmospheric pressure. Chem. Eng. Sci. 2020, 219, 115589 10.1016/j.ces.2020.115589.

[ref9] Quindimil A.; et al. Effect of metal loading on the CO_2_ methanation: A comparison between alumina supported Ni and Ru catalysts. Catal. Today 2020, 356, 419–432. 10.1016/j.cattod.2019.06.027.

[ref10] Tébar-Soler C.; et al. Low-oxidation-state Ru sites stabilized in carbon-doped RuO_2_ with low-temperature CO_2_ activation to yield methane. Nat. Mater. 2023, 22 (6), 762–768. 10.1038/s41563-023-01540-1. 37142737

[ref11] Niu Y.; et al. Manipulating interstitial carbon atoms in the nickel octahedral site for highly efficient hydrogenation of alkyne. Nat. Commun. 2020, 11, 3324 10.1038/s41467-020-17188-3. 32620829 PMC7335178

[ref12] Likith S. R. J.; et al. Thermodynamic Stability of Molybdenum Oxycarbides Formed from Orthorhombic Mo_2_C in Oxygen-Rich Environments. J. Phys.Chem.C 2018, 122 (2), 1223–1233. 10.1021/acs.jpcc.7b11110.

[ref13] Yan C.; et al. Local Built-In Electric Field Enabled in Carbon-Doped Co_3_O_4_ Nanocrystals for Superior Lithium-Ion Storage. Adv. Funct. Mater. 2018, 28, 1705951 10.1002/adfm.201705951.

[ref14] Park Y.; et al. Carbon-doped TiO_2_ photocatalyst synthesized without using an external carbon precursor and the visible light activity. Appl. Catal., B 2009, 91, 355–361. 10.1016/j.apcatb.2009.06.001.

[ref15] Ruano D.; et al. Dynamic Structure and Subsurface Oxygen Formation of a Working Copper Catalyst under Methanol Steam Reforming Conditions: An *in Situ* Time-Resolved Spectroscopic Study. ACS Catal. 2019, 9, 2922–2930. 10.1021/acscatal.8b05042.

[ref16] Ruan F.; et al. Enhanced activity for aerobic oxidative of alcohols over manganese oxides stimulated with interstitial nitrogen doping. Green Synth. Catal. 2021, 2, 38–44. 10.1016/j.gresc.2020.12.003.

[ref17] Jin Y. Jahn-Teller distortion assisted interstitial nitrogen engineering: enhanced oxygen dehydrogenation activity of N-doped Mn_*x*_Co_3–*x*_O_4_ hierarchical micro-nano particles. Nano Res. 2021, 14 (8), 2637–2643. 10.1007/s12274-020-3266-y.

[ref18] Rutt H. N.; Nicola H. J. Raman spectra of carbonates of calcite structure. J. Phys. C: Solid State Phys. 1974, 7, 4522–4528. 10.1088/0022-3719/7/24/015.

[ref19] Buzgar N.; Apopei A. I. The Raman study of certain carbonates. Geologie 2009, 55 (2), 97–112.

[ref20] Koura N.; et al. Alkali carbonates: Raman spectroscopy, ab initio calculations, and structure. J. Mol. Struct. 1996, 382, 63 10.1016/0022-2860(96)09314-3.

[ref21] Wang X.; et al. Kinetic modelling and transient DRIFTS–MS studies of CO_2_ methanation over Ru/Al_2_O_3_ catalysts. J. Catal. 2016, 343, 185–195. 10.1016/j.jcat.2016.02.001.

[ref22] Chen S.; et al. Raising the CO_*x*_ Methanation Activity of a Ru/γ-Al_2_O_3_ Catalyst by Activated Modification of Metal–Support Interactions. Angew. Chem., Int. Ed. 2020, 59, 22763–22770. 10.1002/anie.202007228. PMC7756902 32750196

[ref23] Nielsen N. D.; Jensen A. D.; Christensen J. M. The roles of CO and CO_2_ in high pressure methanol synthesis over Cu-based catalysts. J. Catal. 2021, 393, 324–334. 10.1016/j.jcat.2020.11.035.

