# Supplementary material for: Stabilization of the Active Ruthenium Oxycarbonate Phase for Low-Temperature CO2 Methanation
Source: ACS Catal. 2024 Mar 6;14(6):4290–300. doi: 10.1021/acscatal.3c05679 (PMC10949189; doi:10.1021/acscatal.3c05679)
Supplement: Supplementary file 1 — cs3c05679_si_001.pdf [file cs3c05679_si_001.pdf]

Supporting information

Stabilization of the active ruthenium oxy-carbonate phase for low temperature CO_2 methanation

Carmen Tébar-Soler[a], Vlad Martin Diaconescu[b], Laura Simonelli[b], Alexander Missyul[b], Virginia Perez-Dieste[b], Ignacio Villar-García[b], Daniel Gómez[a], Jean-Blaise Brubach[c], Pascale Roy[c], Avelino Corma [a]*, Patricia Concepción[a]*

[a]*Instituto de Tecnología Química, Universitat Politècnica de València-Consejo Superior de Investigaciones Científicas (UPV-CSIC), Avenida de los Naranjos s/n, 46022 Valencia, Spain*

[b] *CELLS - ALBA Synchrotron Radiation Facility, Carrer de la Llum 2-26, 08290 Cerdanyola del Vallès, Spain*

[c] *Synchrotron SOLEIL, AILES beamline, L'Orme des Merisiers, 91190 Saint Aubin, France.*

* *Instituto de Tecnología Química, Universitat Politècnica de València-Consejo Superior de Investigaciones Científicas (UPV-CSIC), Avenida de los Naranjos s/n, 46022 Valencia, Spain*.

E-mail address: pconcepc@upvnet.upv.es; acorma@itq.upv.es

1. RuO_xC_y@C catalysts S3
1.1. Synthesis of RuO_xC_y@C sample S3
1.2. Catalytic trend S3
1.3. Spectroscopic characterisation of the used samples at 1, 10 and 20 bar S3
1.3.1. X-Ray Diffraction (XRD) S3
1.3.2. Synchrotron X-Ray Diffraction (SXRD) S5
1.3.3. X-ray Absorption Spectra (XAS) S7
1.3.4. Synchrotron X-Ray Photoelectron Spectroscopy (XPS) S16
1.3.5. Synchrotron Far InfraRed studies (FIR) S21
1.4. Catalytic performance S23
1.4.1. Stabilization of the RuO_2C_y with the partial pressure of CO_2 ..... S23
1.4.2. Analysis of Initial reaction rates S23
1.4.3. Influence of the partial pressure of H_2 and CO_2 on the reaction rate and catalyst stability S24
1.4.4. Space velocity and its influence on the catalyst performance S27
1.4.5. CO_2 hydrogenation transient study S28
1.4.6. CO_2 hydrogenation in the presence of trace amount of CO S29
2. Ru^0-RuO_xC_y@C catalysts S32
2.1. Synthesis of Ru^0- RuO_xC_y@C sample S32
2.2. Temperature programme reduction in H_2 (TPR-H_2) S32
2.3. Spectroscopic characterisation of the fresh and used samples at 1, 10 and 20 bar S33
2.3.1. X-Ray Diffraction (XRD) S33
2.3.2. X-ray Absorption Spectra (XAS) S34
2.3.3. Synchrotron X-Ray Photoelectron Spectroscopy (XPS) S38
3. Ru^0-RuO_xC_y@C-200 ..... S42
3.1 Synthesis of Ru^0- RuO_xC_y@C-200 sample S42
3.2. XRD characterisation of the fresh sample S42
4. Hydrogen/Deuterium (H_2/D_2) exchange experiments S43
5. References S45

1. RuO_xC_y@C catalysts

1.1. Synthesis of RuO_xC_y@C sample

RuO_xC_y@C sample is prepared using the synthetic procedure described in ref 10. In detail, the catalyst is synthesized using 120 mg of glucose (Aldrich, > 99.5 %), 7 mL of miliQ water, and 100 mg of RuO_2 (Aldrich, 39 nm). Then, all the chemicals are loaded into a Teflon-coated stainless-steel autoclave of 15 mL and introduced in an oven at 175 °C under static conditions for 24 h. Afterwards, the autoclave is removed from the oven and cooled to room temperature for 2 h. The black solid of the autoclave is filtrated and washed with abundant distilled water and acetone. It is then dried in an oven at 60 °C overnight.

1.2. Catalytic trend

Figure S1 shows the practical impact of the catalyst that has already been reported in our previous work *(10)*. CO_2 hydrogenation is performed at 20 bar, 220°C or 180 °C (indicated in the graph), 1:3 CO_2:H_2 (23.7 % CO_2, 71.3 % H_2, 5 % N_2) and 24000 h^{-1}.

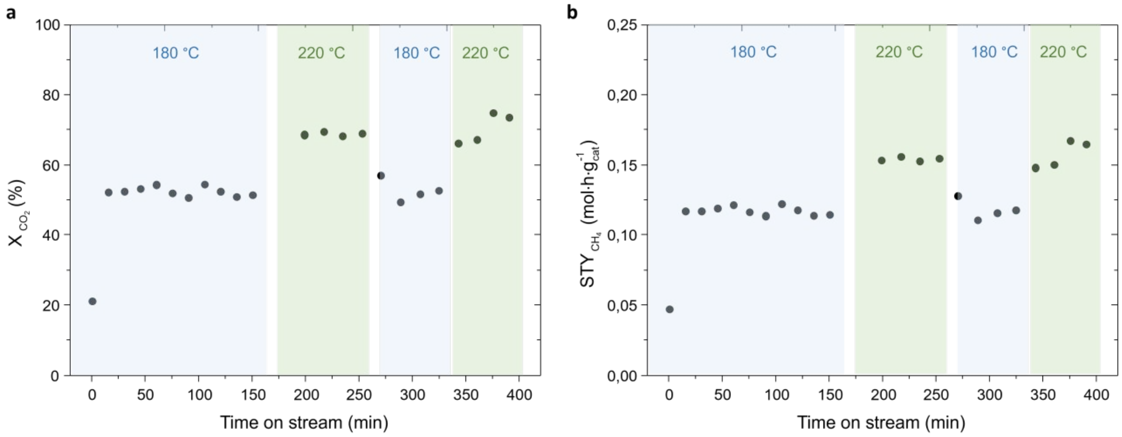

Figure S1. Evolution of CO_2 conversion (left) and methane production (right) with time of stream and different temperatures. Reaction conditions: 20 bar, 100 mL/min, 1:3 CO_2:H_2, and 24000 h^{-1}.

1.3. Spectroscopic characterisation of the used samples at 1, 10 and 20 bar

1.3.1. X-Ray Diffraction (XRD)

Figure S2 shows the characteristic peaks of the Ru^0 (in black), RuO_2 (in red) and RuO_2C_y (in green) phases. The main diffraction peaks together with Miller index of Ru^0 (JCPDS:00-006-0663), RuO_2 and RuO_2C_y (CCDC: 2248295) (*10*) are shown in the Tables S1-S3, respectively.

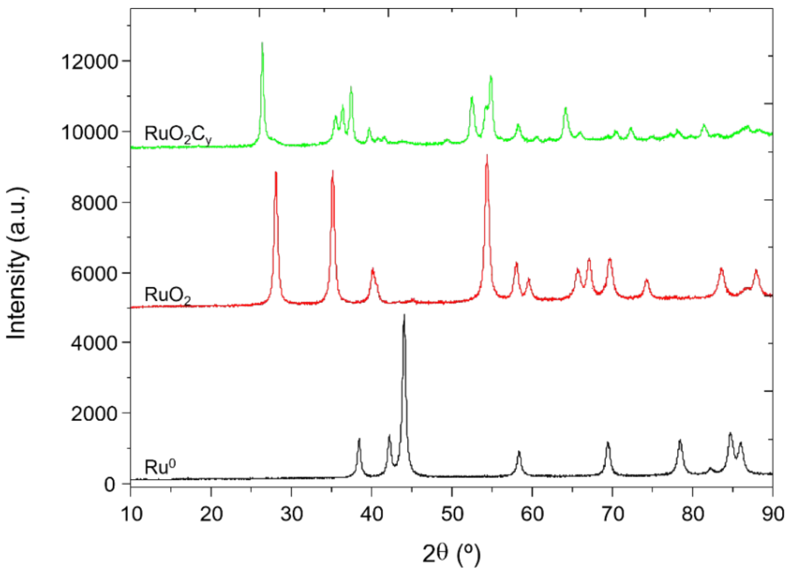

Figure S2. XRD diffraction pattern of Ru_0 (in black), RuO_2 (in red), and RuO_2C_y (in green).

Table S1. Miller index of Ru^0.

h	k	l	2θ (°)
1	0	0	38.39
0	0	2	42.19
1	0	1	44.02
1	0	2	58.35
2	-1	0	69.42
1	0	3	78.41
2	-1	2	84.70
2	0	1	85.95

Table S2. Miller index of RuO_2.

h	k	l	2θ (°)
1	1	0	28.07
1	0	1	35.09
2	0	0	40.12
2	1	1	54.34
2	2	0	58.03
3	1	0	65.68
1	1	2	67.01
3	0	1	69.65
3	2	1	83.54
4	1	1	97.05

Table S3. Miller index of RuO_2C_y (CCDC: 2248295).

h	k	l	2θ (°)
1	0	2	26.29
1	0	-2	26.32
2	0	0	35.40
1	2	0	36.29
0	2	2	37.36
0	0	4	39.60
2	2	2	52.39
2	2	-2	52.43
2	0	4	54.11
2	0	-4	54.18
1	2	4	54.77
1	2	-4	54.81
3	0	2	58.19
3	0	-2	58.24
3	2	0	64.13

1.3.2. Synchrotron X-Ray Diffraction (SXRD)

Data are collected at the ALBA synchrotron Synchrotron Light Facility at the BL-04 MSPD beamline (*25*). The measurements are carried out using an MYTHEN position sensitive detector at 20 keV photon energy (wavelength 0.6201 Å refined using NIST 640d standard). The used samples have been measured ex situ after removal from the reactor and preservation in a N_2 atmosphere. Table S4 shows the composition of the fresh and used samples at different reaction pressure and displayed graphically in Figure S3. These data illustrate that by increasing the reaction pressure, RuO_2C_y is stabilized, inhibiting Ru metal formation (Ru^0).

Table S4. Composition of the fresh and used samples according to SXRD data.

Sample	RuO_2C_y			Ru^0	
	wt (%)	y	Size (nm)	wt (%)	Size (nm)
Fresh	90 ± 1	0.41 ± 0.03	44 ± 5	10 ± 1	4 ± 1
1 bar	0.0	-	-	100.0	13 ± 1
10 bar	45 ± 1	0.51 ± 0.03	40 ± 5	55 ± 1	8 ± 1
20 bar	66 ± 1	0.79 ± 0.03	44 ± 5	34 ± 1	6 ± 1

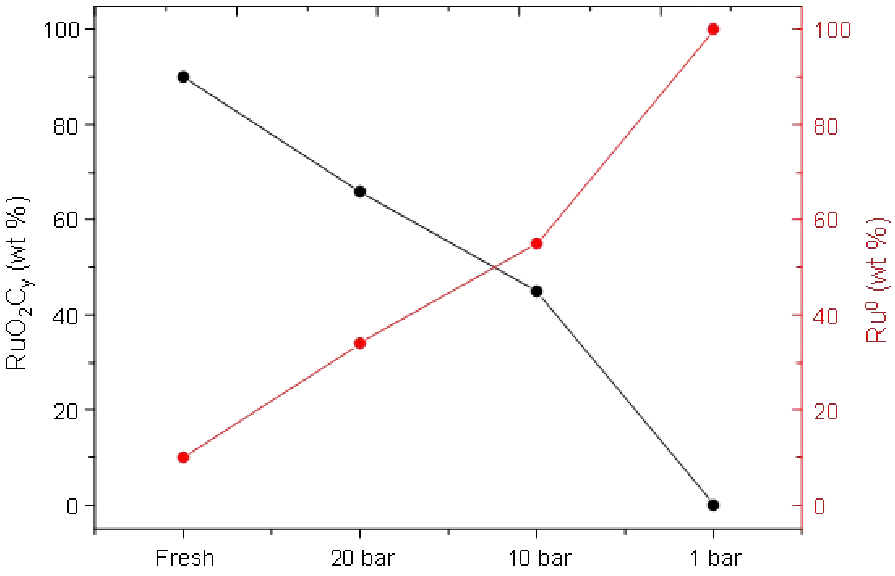

Figure S3. Evolution of RuO_2C_y (left) and Ru^0 (right) fraction on the fresh and used samples at different reaction pressures.

Next, the evolution of the composition of the catalyst with time on stream when operating at 10 bar is shown in Figure S4. The SXRD pattern is obtained on used samples after stopping the reaction at selected reaction times (marked as red circles in the catalytic data of Figure S4). From the corresponding SXRD pattern, it is observed that at 50 min (point 1), the amount of RuO_2C_y and Ru^0 is practically 1:1 (wt %), whereas when deactivation starts (from 100 min onwards) this ratio decreases, increasing the Ru^0 phase.

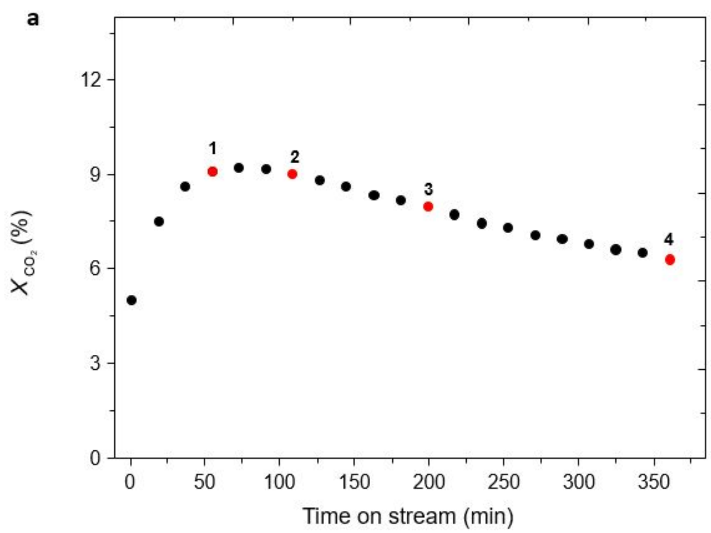

b

Number	Time (min)	RuO_2C_y (%)	Ru (%)
1	50	58 ± 1	42 ± 1
2	100	50 ± 1	50 ± 1
3	200	40 ± 1	60 ± 1
4	360	37 ± 1	63 ± 1

Figure S4. a) CO_2 conversion with the time on stream on the RuO_xC_y@C catalyst at 10 bar. Reaction

conditions: 180 °C, H_2: CO_2 molar ratio 3:1 and GHSV 120000 h^{-1}. b) Phase composition of the used sample according to SXRD by stopping the reaction at different times on stream and operating at 10 bar.

1.3.3. X-ray Absorption Spectra (XAS)

Ru K-edge X-ray Absorption spectra (XAS) are collected at the ALBA synchrotron Synchrotron Light Facility, specifically at BL-22 CLAESS beamline (*26*), as well as at the Advanced Photon Source (APS) 10-BM-A, B beamline of the Argonne National Laboratory for data at 20 bar. The samples are diluted in boron nitride (about 6 mg of sample in 100 mg of boron nitride) and then pelletized. The spectra are collected in transmission mode using a homemade catalytic cell, allowing to record XAS spectra under reaction conditions (1 - 20 bar) and (25 - 200 °C) and connected online to a mass spectrometer (OMNIStar GSD 320 Gas Analysis System from Pfeiffer). In the operando studies, the sample was pelletized as previously described. Then, the cell was pressurized to the desired pressure (i.e., 1 - 20 bar) in He flow (50 mL/min). Once the pressure is achieved, He is exchanged by the reactant feed composed of CO_2 (5 mL/min) and H_2 (15 mL/min). After stabilization, the temperature was increased to 160-180 °C (5 °C/min) and kept at that temperature for 6 hours. After the catalytic process, the system was cooled down to room temperature. XAS and MS were acquired continuously during the thermal treatments and the reaction. . When working with ex-situ data, several XAS repeats are collected (in order to ensure reproducibility and statistics) and the averaged spectra are processed by the Athena software package (*27*). For operando data, the Larch analysis package having the Athena functionality implemented in Python, is employed (*28*). Principal component analysis was carried out in Larch over the XANES region of the spectra from 22100 to 22200 eV, followed by MCR-ALS analysis using the pyMCR (Multivariate Curve Resolution in Python) (*29*) using either RuC_xO_y@C, or Ru^0- RuC_xO_y@C along with Ru^0 and RuO_2 as reference input spectra. MCR-ALS was carried out using Ordinary Least-Squares, with the sum to 1 and non-negativity constraints for the concentrations.

The energy scale is calibrated by adjusting the first inflection point of the metallic Ru spectra at 22117 eV. The EXAFS oscillations are extracted using the autobk algorithm employing a spline in the 0 to 19 $Å^{-1}$ region of the *k*-space having an R_{bkg} of 1, unless otherwise specified. The FEFF6 code (*30-31*) is used for scattering path generation, and multi (k^1, k^2, k^3)-weighted fits of the data are carried out in R-space over the r- and *k*- ranges specified in the text. The S_0^2 value is set to 0.9, and a global E_0 is employed with the initial E_0 value set to the first inflection point of the rising edge. Single scattering paths are fit in terms of a Δreff and σ^2, which represent the deviation from the expected interatomic distances and the structural disorder, respectively. To evaluate the validity of the adjustments, the R_{factor} (%R) and the

reduced χ^2 (χ^2_v) are minimized, ensuring that the data are not over-fit. The best-suited models are determined using a grid search with fixed values for path coordination numbers (N) using Larch, the Artemis Python implementation *(28)*.

Figure S5 shows the EXAFS fits (in black) of data (in green) presented in Table S5, showing overlays in r-space, k-space. Figure S6 shows the XANES rising edge of RuO_xC_y@C (fresh and used after 20, 10 and 1 bar) contrasted to references Ru black (Ru^0) and RuO_2 and the Fourier transformed k^2-weighted EXAFS spectrum. Figure S7 shows the operando XAS data for RuO_xC_y@C under reaction conditions (CO_2+H_2) at 1 bar, 10 bar and 20 bar, as well as at 20 bar under He. Figure S8, shows the evolution of methane formation in the mass spectra of the operando study performed at 1 bar and 10 bar.

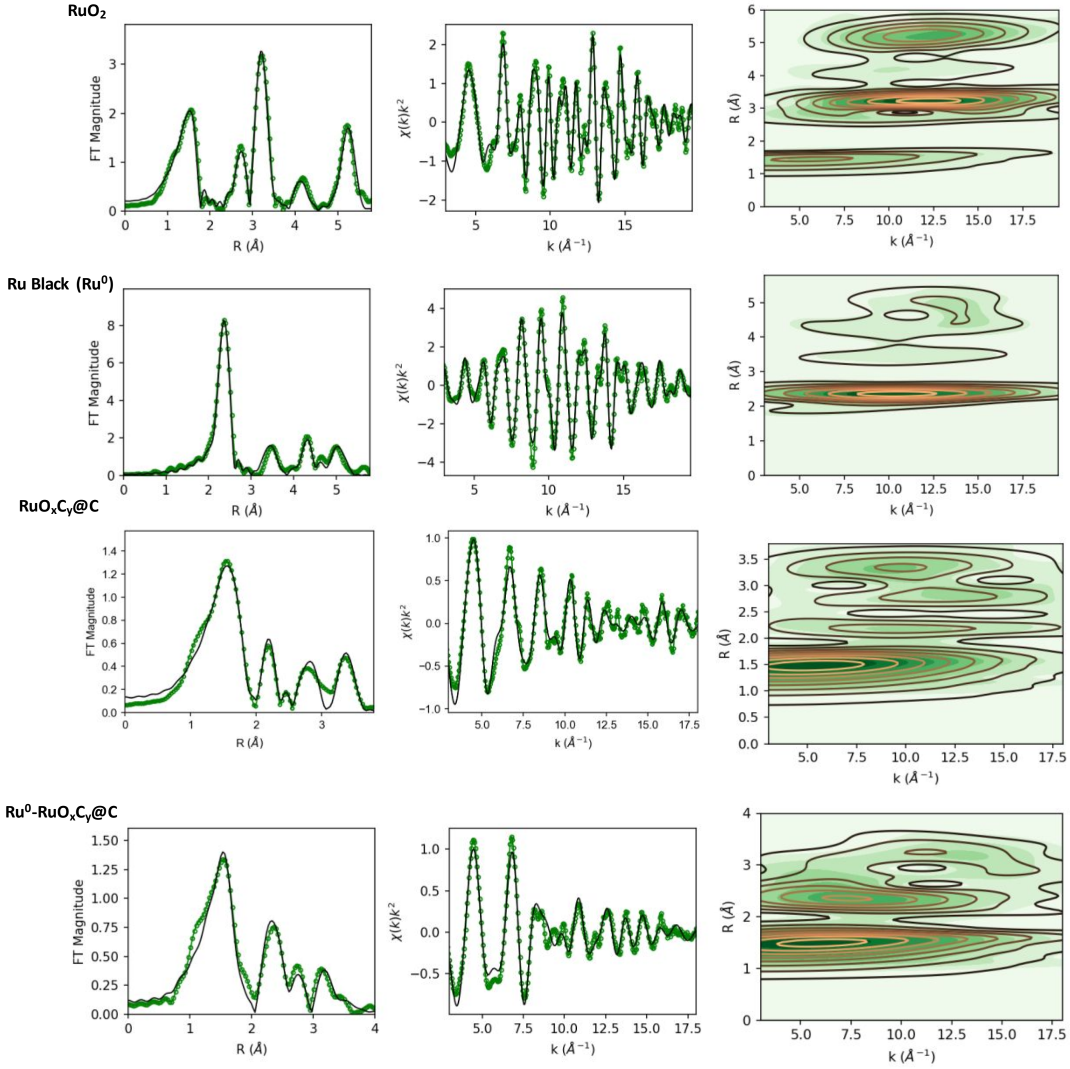

Figure S5: EXAFS fits (black) of data (green) presented in Table S5, showing overlays in r-space, k-space.

Table S5 : Summary of selected EXAFS fits for RuO_2, Ru Black, RuC_xO_y@C, and fresh Ru^0-RuC_xO_y@C catalysts. Multi (k^1, k^2, k^3)-weighted fits carried out in r-space over the ranges indicated using a Hannings window (dk 1), and S_0 = 0.9. Bond distances and disorder parameters (Δr_{eff} and σ^2) were allowed to float having initial values of 0.0 Å and 0.003 Å^2 respectively, with a universal E_0 and ΔE_0 = 0 eV. The errors of the fitting parameters are reported in brackets.

RuO_2

R_{FACTOR}	X^2_v	Var. No.	k-range (Å^{-1})	r-range (Å)	ΔE_0 (eV)
0.038	674	9	3-19.5	1-5.8	-3.6(0.8)

PATHS

Ru - C/N/O			Ru - Ru						Ru - O - Ru - O			Ru - Ru						Ru - Ru - O		
N	r (Å)	σ^2 (x10^3 Å^2)	N	r (Å)	σ^2 (x10^3 Å^2)	N	r (Å)	σ^2 (x10^3 Å^2)	N	r (Å)	σ^2 (x10^3 Å^2)	N	r (Å)	σ^2 (x10^3 Å^2)	N	r (Å)	σ^2 (x10^3 Å^2)	N	r (Å)	σ^2 (x10^3 Å^2)
6.0	1.96(0.01)	2.4(0.4)	2.0	3.11(0.00)	1.5(0.4)	8.0	3.54(0.00)	1.9(0.1)	6.0	3.93(0.02)	2.4(0.4)	4.0	4.48(0.00)	2.8(0.2)	8.0	5.46(0.00)	2.8(0.2)	16.0	5.64(0.00)	2.8(0.2)

Ru Black (Ru^0)

R_{FACTOR}	X^2_v	Var. No.	k-range (Å^{-1})	r-range (Å)	ΔE_0 (eV)
0.032	641	7	3-19.5	1-5.8	-6.1(0.5)

PATHS

Ru - Ru												Ru - Ru - Ru			Ru - Ru			Ru - Ru - Ru - Ru		
N	r (Å)	σ^2 (x10^3 Å^2)	N	r (Å)	σ^2 (x10^3 Å^2)	N	r (Å)	σ^2 (x10^3 Å^2)	N	r (Å)	σ^2 (x10^3 Å^2)	N	r (Å)	σ^2 (x10^3 Å^2)	N	r (Å)	σ^2 (x10^3 Å^2)	N	r (Å)	σ^2 (x10^3 Å^2)
12.0	2.67(0.00)	2.8(0.1)	6.0	3.79(0.00)	2.7(0.3)	12.0	4.66(0.00)	2.7(0.3)	12.0	5.07(0.01)	4.0(0.5)	24.0	5.19(0.01)	4.0(0.5)	12.0	5.43(0.01)	4.0(0.5)	6.0	5.43(0.01)	4.0(0.5)

RuO_xC_y@C

R_{FACTOR}	X^2_v	Var. No.	k-range (Å^{-1})	r-range (Å)	ΔE_0 (eV)
0.020	425	10	3-18	1-3.8	-2.1(1.0)

PATHS

Ru - C/N/O						Ru - Ru																	
N	r (Å)	σ^2 (x10^3 Å^2)	N	r (Å)	σ^2 (x10^3 Å^2)	N	r (Å)	σ^2 (x10^3 Å^2)	N	r (Å)	σ^2 (x10^3 Å^2)	N	r (Å)	σ^2 (x10^3 Å^2)	N	r (Å)	σ^2 (x10^3 Å^2)	N	r (Å)	σ^2 (x10^3 Å^2)	N	r (Å)	σ^2 (x10^3 Å^2)
2.0(0.2)	1.97(0.01)	3.2(0.6)	3.0(0.6)	2.06(0.01)	3.2(0.6)	0.6(0.1)	2.54(0.01)	1.5(0.5)	1.2(0.1)	2.66(0.01)	4.5(0.8)	1.2(0.2)	2.80(0.01)	10.0(0.1)	0.6(0.1)	3.11(0.01)	1.5(0.5)	0.6(0.1)	3.17(0.01)	10.0(0.1)	2.4(0.5)	3.69(0.01)	4.5(0.8)

Ru^0-RuO_xC_y@C

R_{FACTOR}	X^2_v	Var. No.	k-range	r-range	ΔE_0
0.027	695	11	3-18	1-4.0	-5.0(1.0)

PATHS

Ru - C/N/O						Ru - Ru											
N	r (Å)	σ^2 (x10^3 Å^2)	N	r (Å)	σ^2 (x10^3 Å^2)	N	r (Å)	σ^2 (x10^3 Å^2)	N	r (Å)	σ^2 (x10^3 Å^2)	N	r (Å)	σ^2 (x10^3 Å^2)	N	r (Å)	σ^2 (x10^3 Å^2)
2.4(0.4)	1.96(0.01)	1.1(0.8)	1.8(0.8)	2.10(0.01)	0.4(1.2)	3.6(0.5)	2.67(0.01)	8.7(0.8)	0.4(0.1)	3.11(0.01)	1.0(1.2)	1.6(0.4)	3.55(0.01)	4.1(1.3)	1.8(0.3)	3.82(0.01)	10.7(6.7)

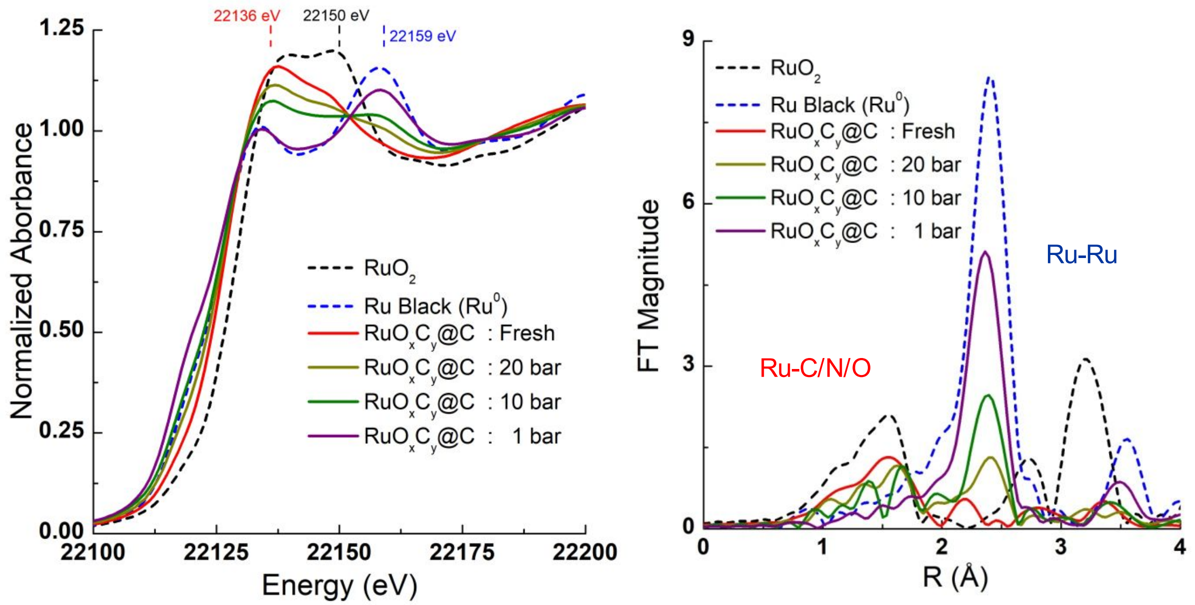

Figure S6. (Left), XANES rising edge of $RuO_xC_y@C$ (fresh and used after 20, 10 and 1 bar) contrasted to references Ru black (Ru^0) and RuO_2. Energies at 22136 eV, 22150 eV and 22159 eV best correlate to RuO_2C_y (red line), RuO_2 (black line) and Ru Black (Ru^0, blue line), respectively. (Right), Fourier transformed k^2-weighted EXAFS spectrum with a k-range of 3-18.9 $Å^{-1}$ and a Hannings window; formation of Ru^0 is observed at 2.4 Å in r-space and is favoured in the samples at 1 bar over that at 10 bar, with 20 bar being the least favoured.

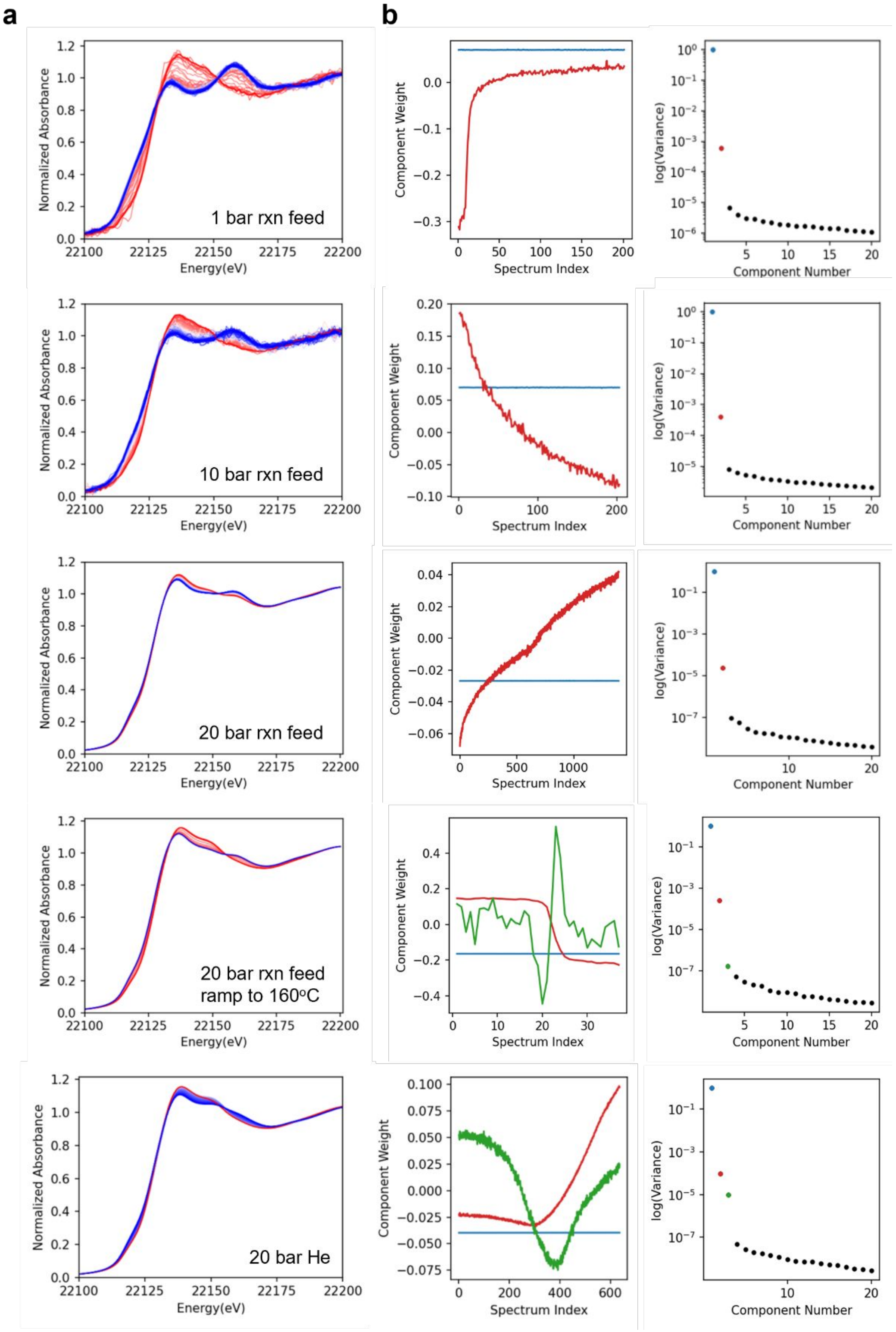
a
b
1 bar rxn feed
10 bar rxn feed
20 bar rxn feed
20 bar rxn feed ramp to 160°C
20 bar He
Normalized Absorbance
Energy(eV)
Component Weight
Spectrum Index
log(Variance)
Component Number

c

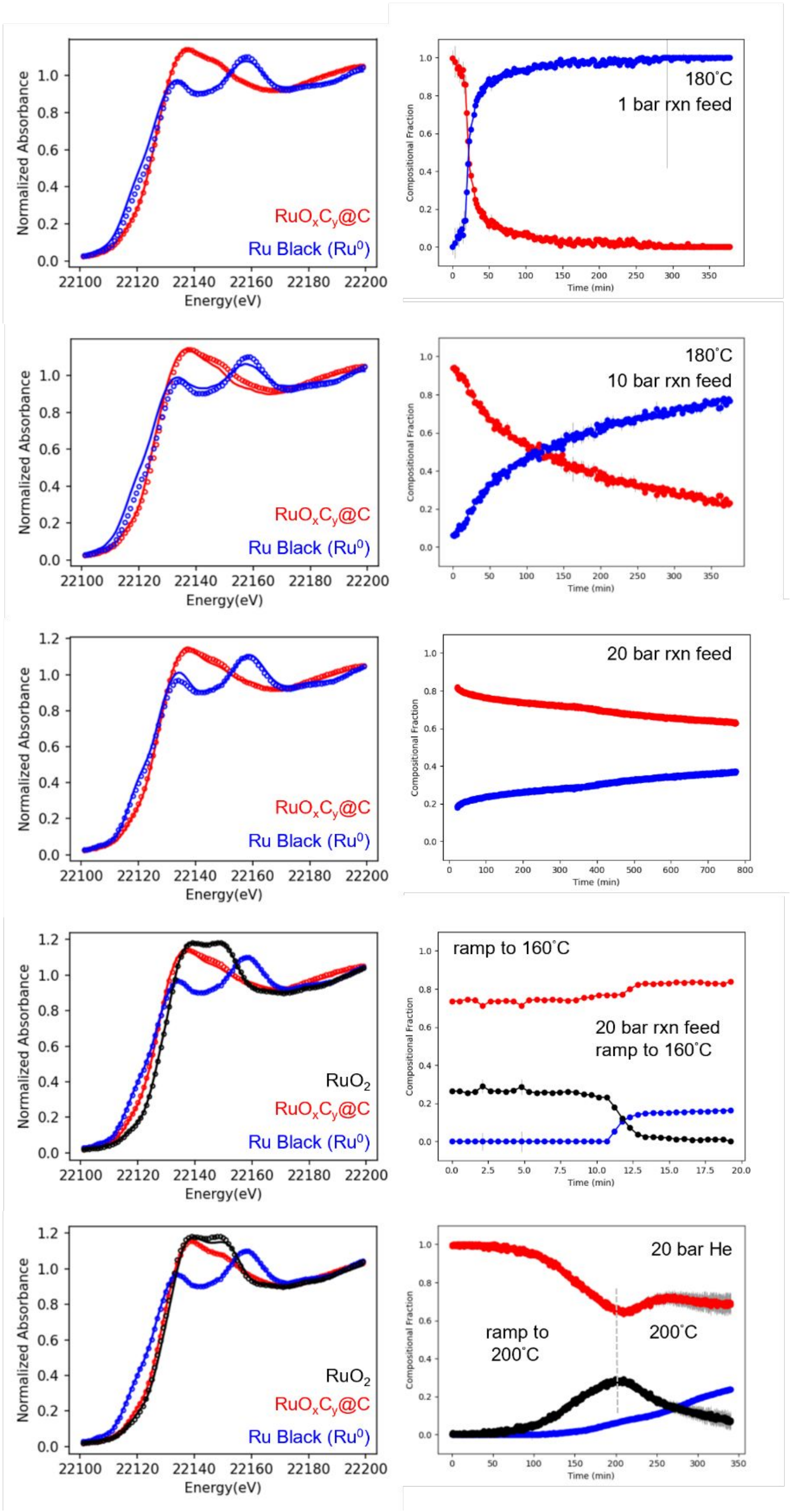
Normalized Absorbance
Energy(eV)
RuO_xC_y@C
Ru Black (Ru^0)
RuO_2
Compositional Fraction
Time (min)
180˚C
1 bar rxn feed
180˚C
10 bar rxn feed
20 bar rxn feed
ramp to 160˚C
20 bar rxn feed
ramp to 160˚C
20 bar He
ramp to
200˚C
200˚C

d

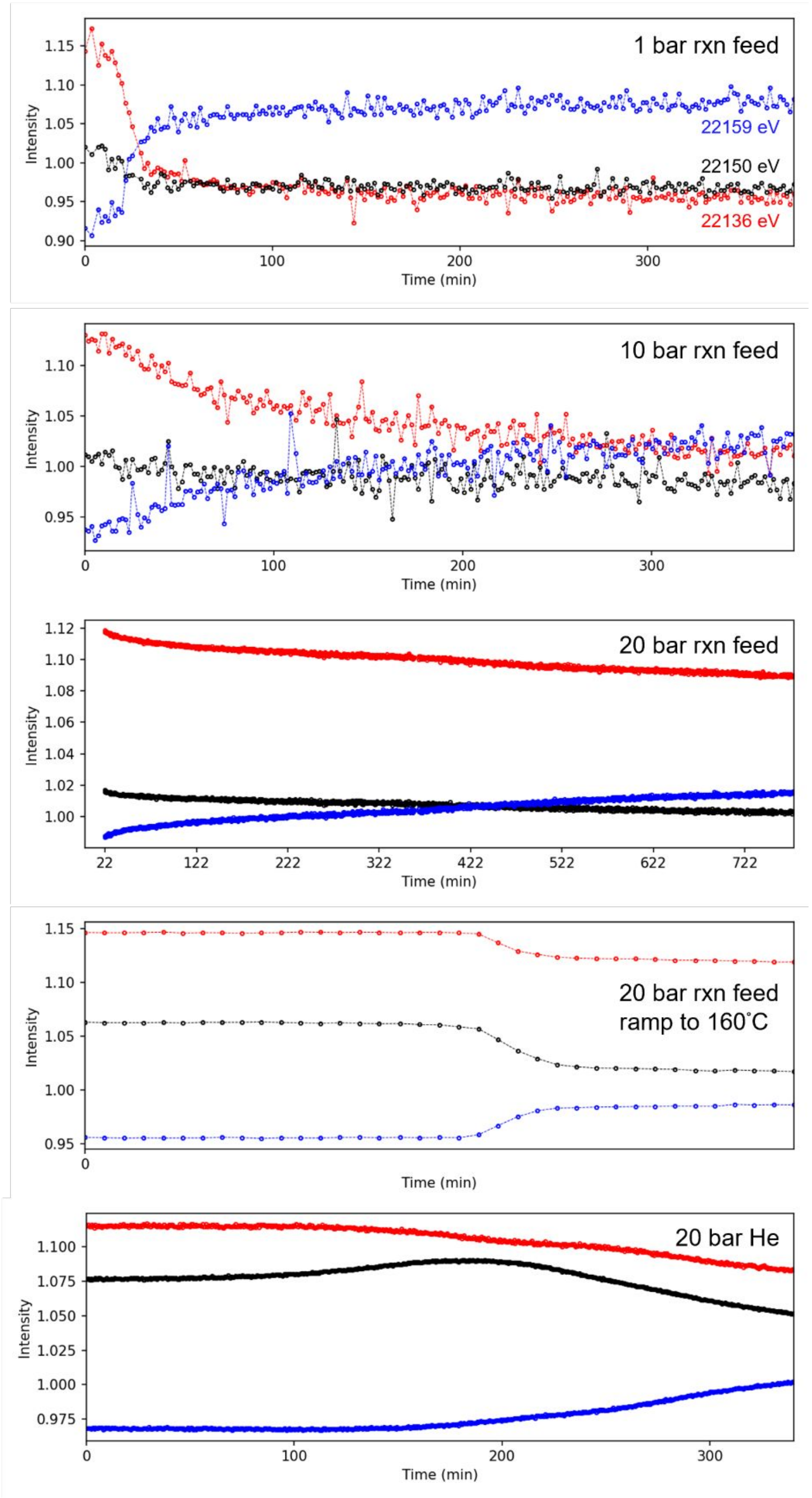

Figure S7. Operando XAS data for RuO_xC_y@C under reaction conditions (CO_2+H_2) at 1 bar, 10 bar and 20 bar as well as at 20 bar under He: (a) XANES of operand data; (b) PCA derived, eigenvectors and scree plot;

(c) MCR-ALS analysis (*16*) showing the input guess component spectra (dots) output determined optimized spectra (lines) and on bottom evolution of fractional contribution over time; Spectra were optimized using Ordinary Least-Squares, with the sum to 1 and non-negativity constraints for the concentrations; (d) Intensity traces at 22136 eV (red), 22150 eV (black) and 22159 eV (blue) that best correlate to RuO_2C_y, RuO_2 and Ru Black (Ru^0) respectively.

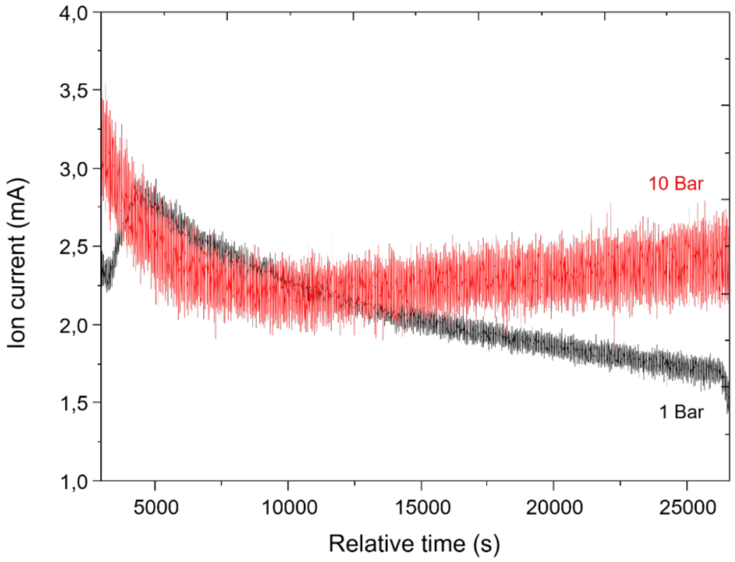

Figure S8. Evolution of methane formation by online mass spectrometry operating at 1 and 10 bar. Methane has been followed using the mass signal m/z=15.

1.3.3.1. Kinetics rates of RuO_2Cy disappearance

The time evolution of the RuO_2Cy component under the reaction conditions displayed in Figure S9 are analysed in order to determine the kinetics of catalyst deactivation. The data are fitted with a linear regression to the Equations (1-3), which describe the different deactivation orders calculated from the general expression $-da/dt = kA^{\mathrm{d}}$, where k represents the deactivation constant and d the deactivation order (i.e., Equation 1 for $\mathrm{d} = 0$, Equation 2 for $\mathrm{d} = 1$, and Equation 3 for $\mathrm{d} = 2$) *(32)*. Figure S9 shows different fittings depending on the velocity order for the evolution of the RuO_2C_y phase to Ru^0 obtained by XAS under different reaction conditions (i.e., 20, 10 and 1 bar). The analysed data are between ~15 min and 120 min for the experiments done at 1 bar (Fig S9a), between ~15 min and 300 min for the experiments at 10 bar (Fig S9b), and between 0 min and 775 min for the ones at 20 bar (Fig S9c).

$$[A]_t = -kt + [A]_0 \#(1)$$

$$Ln[A]_t = -kt + Ln[A]_0 \#(2)$$

$$1/[A]_t = -kt + 1/[A]_0 \#(3)$$

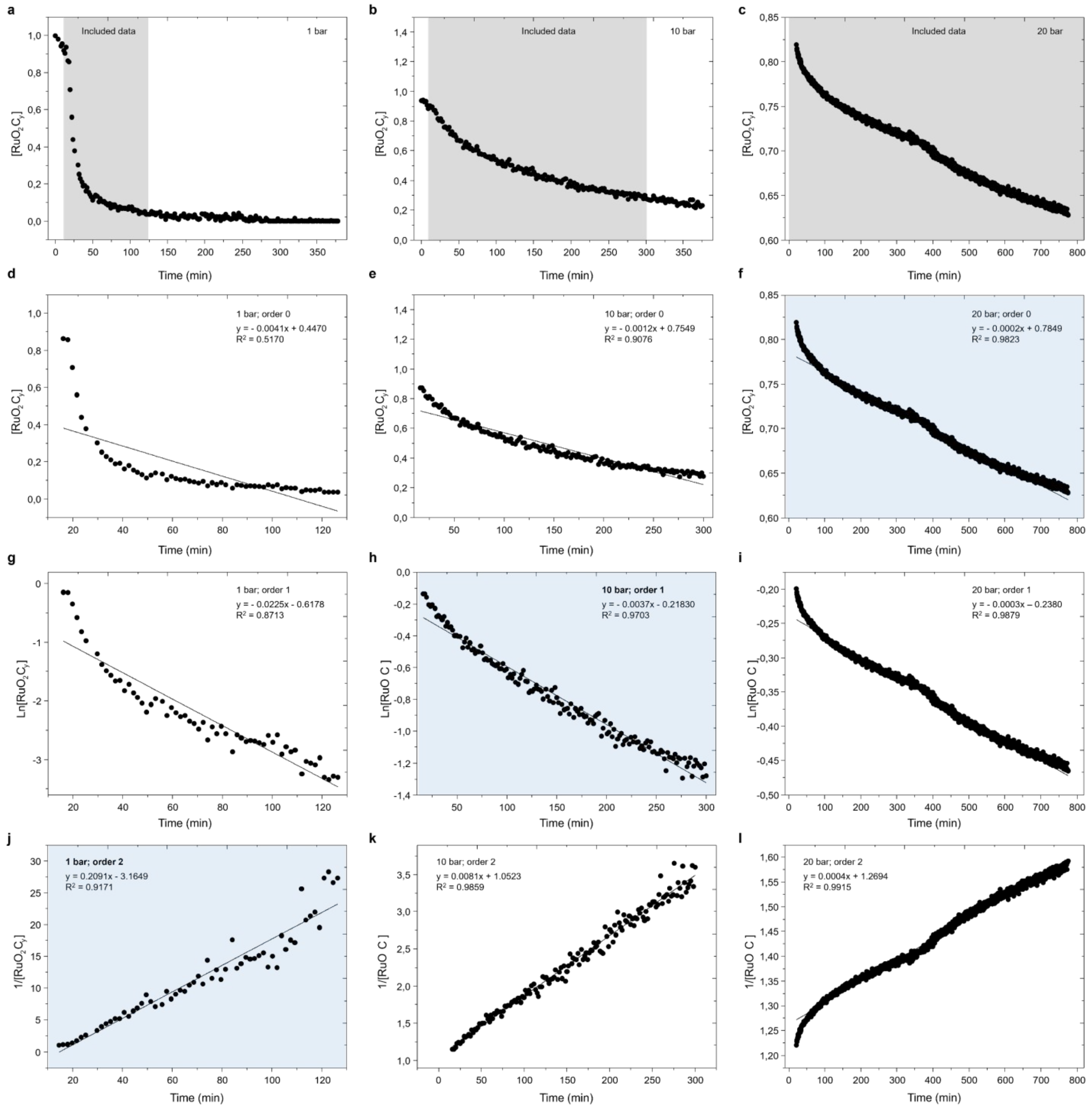

Figure S9. (a-c) Evolution of the RuO_2C_y phase with time at different pressures: (a) 1, (b) 10, and (c) 20 bar. The grey zone corresponds to the data that are used for the kinetics fittings. (d-f) lineal regression of the evolution of the RuO_2C_y phase with time at the different reaction conditions: 1 (d), 10 (e) and 20 (f) bar,

corresponding to a cero order equation. (g-i) Lineal regression of the evolution of Ln(RuO_2C_y) with time at the different reaction conditions: 1 (d), 10 (e) and 20 (f) bar, corresponding to a one order equation. (j-l) Lineal regression of the evolution of 1/(RuO_2C_y) with time at the different reaction conditions: 1 (d), 10 (e) and 20 (f) bar, corresponding to a second order equation. The blue graphs correspond with the better linear fitting (i.e., cero order for the processes at 20 bar, one order at 10 bar and second order for the processes at 1 bar). The results show that the deactivation phenomena is related to the pressure and agree with SRXD data in which at 1 bar only Ru^0 appears due to higher RuO_2C_y reduction.

1.3.4. Synchrotron X-Ray Photoelectron Spectroscopy (XPS)

Synchrotron XPS experiments are performed in beamline BL-24 (CIRCE) at the ALBA Synchrotron Light Facility. The end station is equipped with a Phoibos 150 NAP electron energy analyser (SPECS GmbH). The beam size is 100 × 30 (height × volume) μm^2, with a pass energy of 10 eV, a step of 0.1 eV and a beamline exit slit of 20 µm. Incident photon energies of 500 and 1.400 eV for Ru 3d and C 1s are used resulting in sample depths of 1.9 and 5.6 nm, respectively. The probing depth is obtained using the QUASES-IMFP-TPP2M software (http://www.quases.com/home/), which is based on the Tanuma Powell and Penn algorithm (TPP2M), to calculate the inelastic mean free path for the RuO_2 model (*33-34*). About 30 mg of sample is pelletized, mounted onto a stainless-steel sample holder, and transferred to the analysis chamber. The spectra are acquired in vacuum (6×10^{-10} mbar) at 25 °C without prior sample activation. Data analysis is performed using Casa XPS software and a Shirley line as the background. For the ruthenium core levels, a tail-dampened Lorentzian asymmetric line shape (LF (α, β, ω, m)) is used, where α and β define the spread of the tail at each side of the Lorentzian component, ω determines the tail-dampening parameter and m is the width of the Gaussian used to convolute the Lorentzian curve. In particular, values of LF (0.8, 1.25, 500, 180) for Ru^0 and LF (0.25, 1, 45, 280) for RuO_2 are used (*35*). For C 1s, a Gaussian (70%)–Lorentzian (30%) curve, defined as GL(30) is used. Spectra are calibrated with respect to C 1s settled at 284.5 eV, and quantitative data are calculated using the cross-sections tabulated in Yeh and Lindau (*36*).

Synchrotron XPS spectra obtained of the fresh and used samples at 1, 10 and 20 bar are given in Fig. S10 and summarized in Table S6 and Figure S11. The used samples have been measured ex situ after removal from the reactor and preservation in a N_2 atmosphere. Core line C 1s + Ru 3d analysis are performed at two different sample depths of 1.9 and 5.6 nm, corresponding to X-ray excitation energies of 500 and 1400 eV, respectively. Deconvolution of the C 1s + Ru 3d peak shows different ruthenium phases at 280.3 (Ru^{n+} in low oxidation state, which corresponds to RuO_2C_y), 280.1 eV a degraded RuO_2C_y phase due to

carbon or oxygen loss (labelled as RuO_2C_y*), 280.8 (Ru (IV) in RuO_2), 281.5 (hydrated, amorphous RuO_2 or Ru (VI) in RuO_3,), 282.4 (satellite of Ru (IV) in RuO_2 (i.e., peak at 280.8)), 279.8 (Ru^0) eV (*9, 37-39*). On the other hand, various components corresponding to hydroxyl (285.5 eV), carbonyl (287.1 eV), and carbonate species (288.7 eV) are also fitted in the C1s peak.

Table S6. Synchrotron XPS of the C 1s and Ru $3d_{5/2}$ core levels on fresh and used samples after 20, 10 and 1 bar. BE (eV) of each component analyzed at two X-ray excitation energy: 500 eV (i.e., depth 1.9 nm) and 1400 eV (i.e., depth 5.6 nm). The numbers in brackets represent the total surface atomic percentage of the components.

Sample	Depth	C 1s				Ru $3d_{5/2}$					C : Ru (atom ratio)
		C-C,C-H	C-OH	RHC=O	RCOOH/ R-O-C(=O)-O-R'	Ru^0	RuO_2C_y	Ru^{IV}	s.$Ru^{IV,a}$	Ru^{VI}	
RuO_xC_y@C	1.9 nm	284.5 *(38.5)*	285.5 *(42.5)*	287.1 *(12.0)*	288.7 *(5.0)*	-	**280.3** *(0.5)*	280.8 *(0.7)*	282.4 *(0.5)*	281.6 *(0.2)*	98.1 : 1.9
	5.6 nm	284.5 *(64.0)*	285.5 *(22.4)*	287.4 *(8.4)*	-	280.0 *(0.4)*	280.4 *(1.9)*	280.9 *(2.1)*	282.3 *(0.8)*	-	94.8 : 5.2
RuO_xC_y@C 20 bar	1.9 nm	284.5 *(14.6)*	284.9 *(47.5)*	286.1 *(25.8)*	288.4 *(6.1)*	279.8 *(0.6)*	280.3 *(2.2)*	280.8 *(1.3)*	282.9 *(1.4)*	281.9 *(0.5)*	94.0 : 5.9
	5.6 nm	284.5 *(35.2)*	285.4 *(46.5)*	287.8 *(4.3)*	-	279.8 *(2.6)*	280.2 *(4.5)*	280.7 *(4.3)*	282.4 *(2.6)*	-	85.9 : 14.0
RuO_xC_y@C 10 bar	1.9 nm	284.1, 284.5, 285.1 *(11.9), (27.6), (19.9)*	285.9 *(26.1)*	-	288.2 *(7.5)*	279.7 *(2.0)*	280.3 *(3.0)*	281.0 *(0.2)*	282.6 *(0.7)*	281.7 *(0.9)*	93.1 : 6.9
	5.6 nm	284.5 *(68.7)*		286.3 *(16.8)*		279.5 *(5.3)*	280.0 *(5.2)*	280.5 *(2.4)*	282.1 *(0.6)*	281.4 *(0.9)*	85.5 : 14.4
RuO_xC_y@C 1 bar	1.9 nm	284.5 *(39.3)*	285.2 *(26.9)*	286.3 *(18.1)*	287.6, 289.1 *(7.2), (3.5)*	279.8 *(1.3)*	**280.1** *(2.4)*	280.8 *(0.2)*	282.7 *(0.61)*	281.8 *(0.61)*	95.0 : 5.0
	5.6 nm	284.5, 285.1 *(34.2), (24.8)*	285.7 *(17.9)*	-	287.4 *(10.2)*	279.7 *(5.8)*	280.2 *(4.3)*	280.9 *(1.5)*	282.1 *(1.4)*	-	87.0 : 12.9

a) s.Ru^{IV}= Satellite of Ru^{IV}

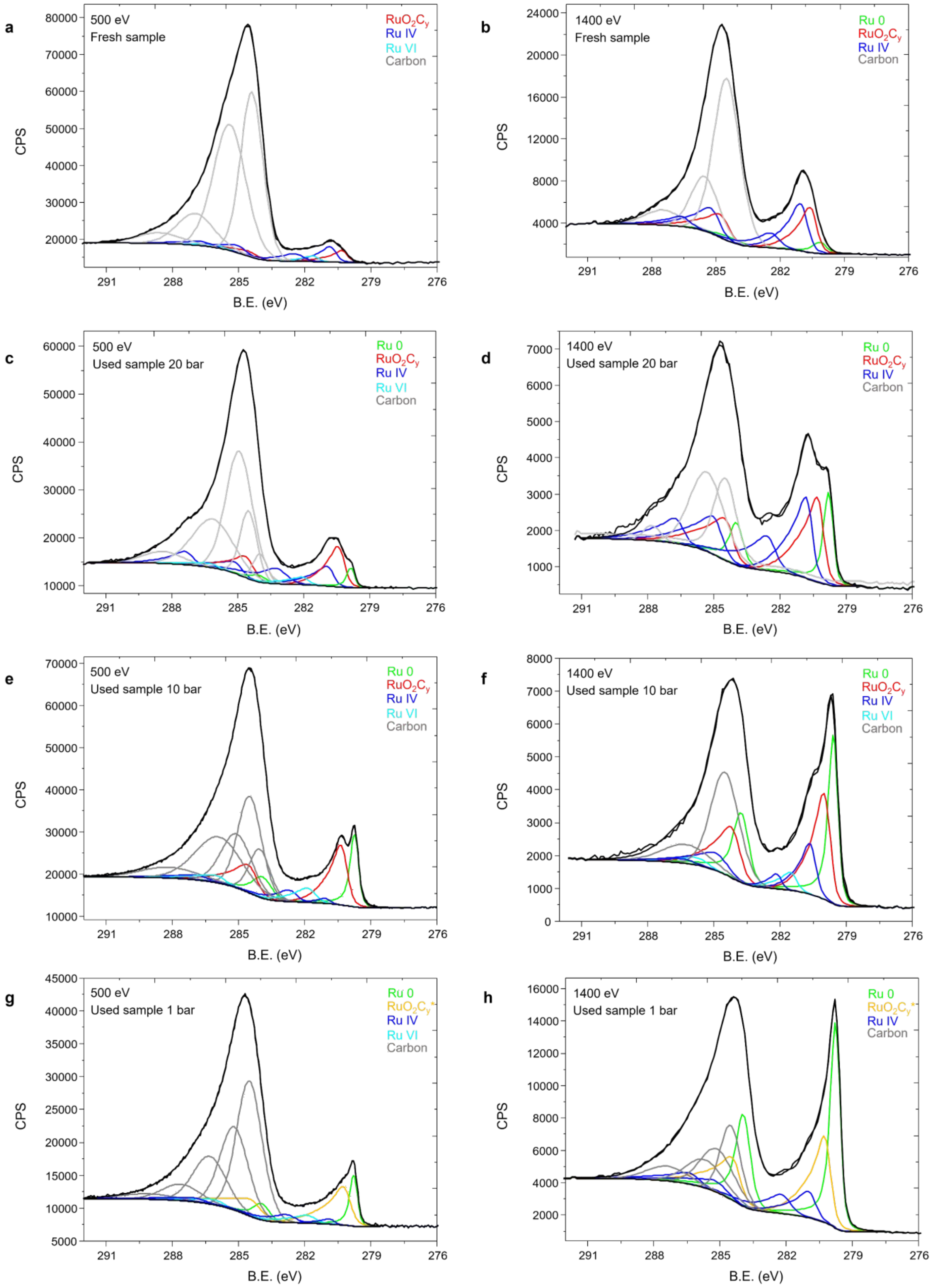

Figure S10. Synchrotron XPS of the C 1s and Ru 3d core levels on fresh (a, b) and used samples after 20 (c, d), 10 (e, f) and 1 (g, h) bar. X-ray excitation energy is 500 eV (i.e., depth 1.9 nm) for the left spectra, and

1400 eV (i.e., depth 5.6 nm) for right spectra. Carbon in grey, RuO_2C_y (in red), metallic ruthenium (labelled as Ru^0, in green), oxidized ruthenium (labelled as Ru IV and Ru VI in dark blue and cyan, respectively) and a degraded RuO_2C_y phase due to carbon or oxygen loss, labelled as RuO_2C_y* (in orange).

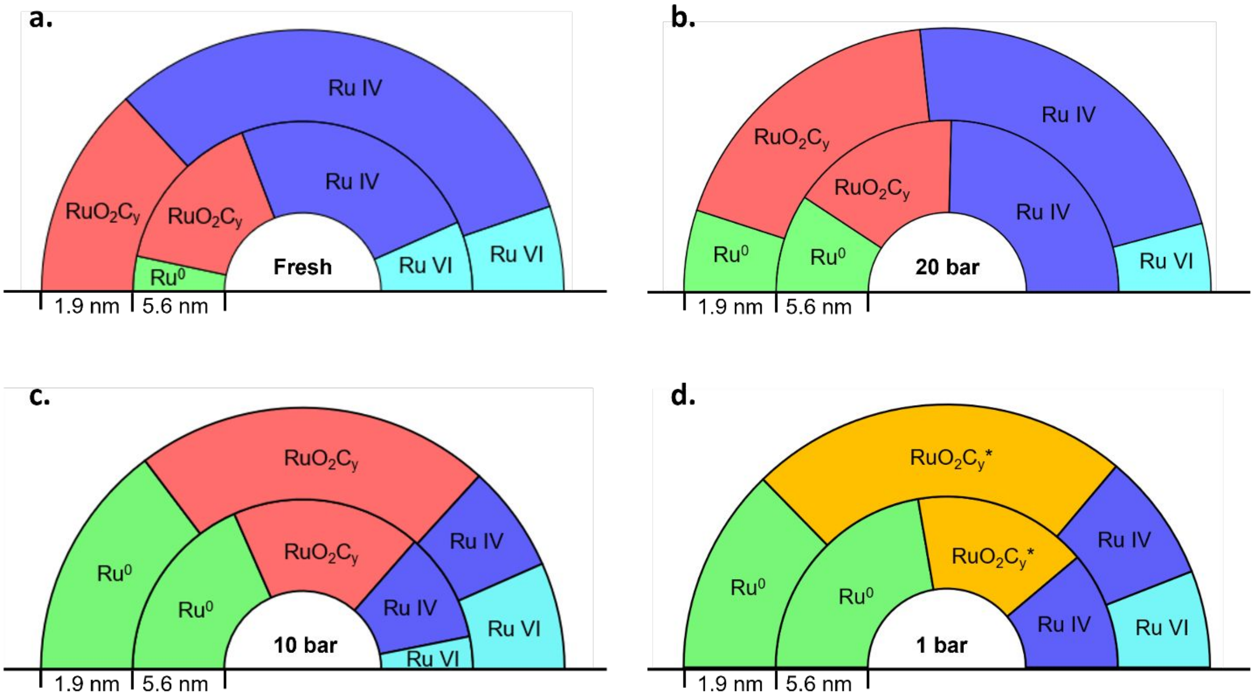

Figure S11. Atomic fraction of the different ruthenium species in: (a) fresh, (b) used sample at 20 bar, (c) used sample at 10 bar, and (d) used sample at 1 bar. The semicircles show the atomic fraction of ruthenium oxy-carbonate (labelled as RuO_2C_y (in red), a degraded RuO_2C_y phase due to carbon or oxygen loss, labelled as RuO_2C_y* (in orange), metallic ruthenium (labelled as Ru^0, in green), and oxidized ruthenium (labelled as Ru IV and Ru VI in dark blue and cyan, respectively). The outer semicircle corresponds to the atomic fraction obtained at 1.9 nm depth ($h\nu$ = 500 eV). The inner semicircle corresponds to the atomic fraction obtained at 5.6 nm depth ($h\nu$ = 1400 eV).

1.3.5. Synchrotron Far InfraRed studies (FIR)

Synchrotron IR spectra were performed at the AILES Beamline of SOLEIL Synchrotron Light Facility (Saint Aubin, Paris, France) (*40*). The IR spectra were acquired using a Bruker IFS 125 MR spectrometer. The experiment was carried out using a homemade cell, recording infrared reflection-absorption spectra under various catalytic conditions of pressure (up to 30 bar) and/or temperature (up to 900°C) and with a direct analysis of the reaction product by online gas chromatography using a MicroGC Fusion from INFICON equipped with two columns (Rt-Q-Bond and Rt-Molsieve 5A) and a MEMS-based micro thermal conductivity detector (microTCD). In the operando studies, the sample holder of the cell was filled with 25 mg of Carbon (Norit) at the bottom and 35 mg of sample on top. Next, a reactant feed composed of CO_2 (7.5 mL/min) and H_2 (22.5 mL/min) was added to the cell. After stabilization, the temperature was increased to 120 °C and 180 °C (5 °C/min) and kept at the final temperature for 6 hours. After the catalytic process, the system was cooled down to room temperature. IR spectra and CG were acquired continuously during the thermal treatments and the reaction. Spectra were recorded in the far infrared region (20 cm^{-1} to 600 cm^{-1}) by using a Si-Mylar multilayer beam splitter, synchrotron source and 4.2K bolometer. Each single spectra were obtained by averaging 500 scans with a resolution of 2 cm^{-1} and at 2cm/s mirror speed. Background spectra were acquired before each experiment, they correspond to the reflectance of a sheet of silver at different pressures and temperatures of H_2O gas. This makes it possible to subtract from each sample spectrum the pure rotational band of gaseous water, produced during the conversion reaction of CO_2 into CH_4, and which is observed in the far infrared range. All spectra are displayed in absorbance units as −log(I/I0), where I and Io are the sample and the background reflection-absorbance spectra, respectively. Data treatments were done with Igor Pro 9 software.

Figure S12 represents the in-situ far infrared spectra of a fresh sample of RuOxCy@C under 1 bar of pressure of the H_2/CO_2 mixture at 120 °C, 180 °C and during the cooling of the reactor to 25 °C after the reaction. The CO_2 conversion is shown in the upper right corner. The 5 spectra shown in Figures S12-A, B and C were measured over a period of approximately 10 minutes. We can observe two bands around 500 cm^{-1} associated with Ru-O (*41*) and the bands at 278 and 375 cm^{-1} associated with the vibration ν(Ru-0-C) of the Ru-CO_3 vibratory group. This series of bands are temperature sensitive and reaches its maximum intensity at 120 °C. They are linked to the interstitial C reconstructed under the conditions of reaction with the CO_2 of the feed. However, at higher temperature (i.e., 180 °C), they do not evolve with the same high temperature trend over time. The bands around 500 cm^{-1} remain almost unchanged, while the 278 and 375 cm^{-1} bands decrease regularly over time (10 % in area of the bands or 11 % in intensity). The reaction

pressure is limited to 1 bar, the reduction of these two bands can be linked to a partial degradation of the structure due to the elimination of carbon. The gradual decrease of the Ru-CO_3 bands in about 10 minutes agrees with the decrease of XANES signal of the RuO_2C_y phase at similar temperature and pressure conditions. This evolution is not observed for the spectra measured at 120 ° C under the same pressure and during the cooling of the sample after the reaction.

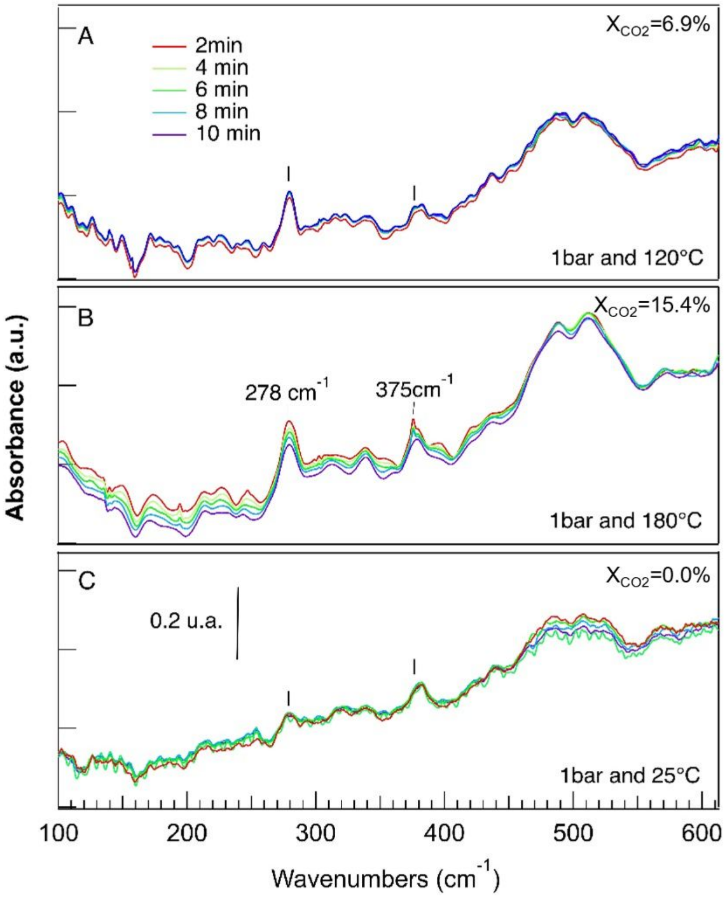

Figure S12. *In-situ* FIR spectra of RuO_xC_y@C sample at 1 bar and different temperatures: (A) 120 °C, (B) 180 °C, and (C) 25 °C (after the cool down the reactor). After stabilization of the temperature 5 spectra were measured at 2 minutes interval. The CO_2 conversion is indicated in the upper right corner.

1.4. Catalytic performance

1.4.1. Stabilization of the RuO_2C_y with the partial pressure of CO_2

CO_2 hydrogenation is carried out in the CO_2 hydrogenation reactor set-up described in ref 10. The standard reaction conditions used in our work are 20 bar, 180 °C and GHSV (space velocity) 120000 h^{-1}, allowing to operate under differential conditions (i.e., conversion <10 %). These conditions are kept for 362 min.

In this set of experiments, the CO_2:H_2 molar ratio is maintained constant at 1:4, but using different partial pressures of CO_2. Black line, labelled as CO_2:H_2 1:4, which corresponds to a feed composition of 19 vol % CO_2, 76 vol % H_2, 5 vol % N_2, and red line, labelled as CO_2:H_2 1:4 diluted, corresponding to 5 vol % CO_2, 20 vol % H_2, 75 vol % N_2. In this sense, the partial pressure of CO_2 is varied between 3.8 (CO_2:H_2 1:4) and 1 bar (CO_2:H_2 1:4 diluted).

As shown in Fig. S13, although maintaining the CO_2:H_2 ratio equal to 4, the catalyst deactivates at operating conditions of low CO_2 partial pressure.

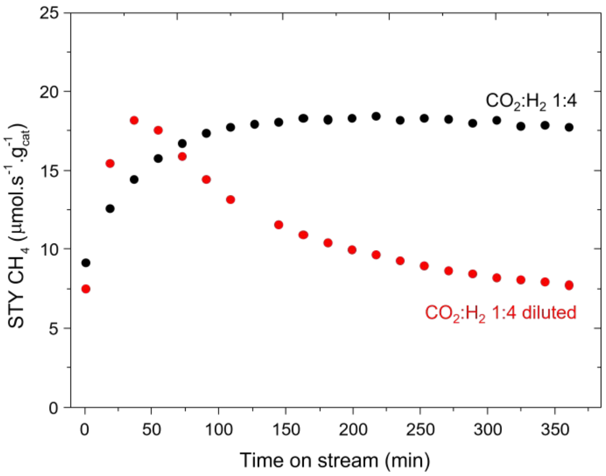

Figure S13. Variation of the yield to methane with the time on stream on the RuO_xC_y@C catalyst at 180 °C, 20 bar, GHSV 120000 h^{-1} and different CO_2 concentrations in the reactant feed. CO_2:H_2 1:4 (19 vol % CO_2, 76 vol % H_2, 5 vol % N_2) (black line) and CO_2:H_2 1:4 diluted (5 vol % CO_2, 20 vol % H_2, 75 vol % N_2) (red line).

1.4.2. Analysis of Initial reaction rates

Initial reaction rates are determined by analyzing the CO_2 conversion at different contact times (W/F). CO_2 hydrogenation takes place at 20 bar, 180 °C, and CO_2:H_2= 1:3. The inlet gas mixture was modified by

adjusting the total flow to modify the contact time. The contact time was varied from 0.9 to 1.5 min·mg·mL^{-1}. Each of these conditions were kept for 63 min.

Fig. S14 shows the variation of the CO_2 conversion with the contact time, resulting in a straight line. The conversion response factor as a function of space velocity in the range of study (i.e., from 0 to 1.5 mg·min·ml^{-1}) is 14.8 mL·mg^{-1}·min^{-1}, which corresponds with an initial rate of methane production of 20.3 µmol·g^{-1}·s^{-1} (assuming 99.9% selectivity to methane).

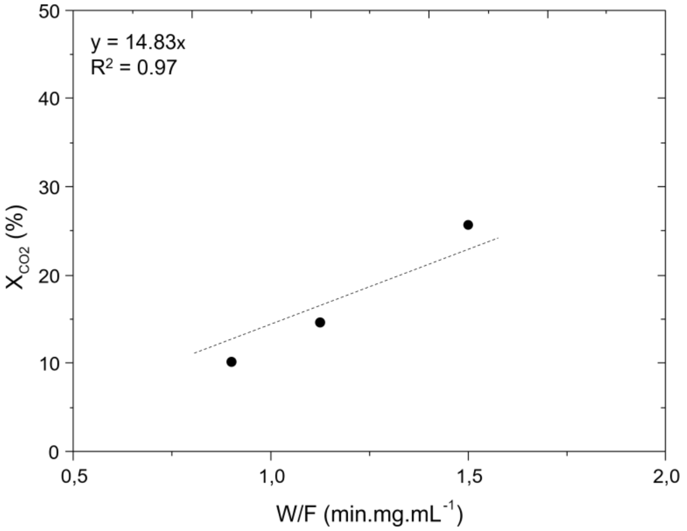

Figure S14. Variation of the contact time with the conversion. Reaction conditions: 20 bar, 180 °C, and CO_2:H_2= 1:3.

1.4.3. Influence of the partial pressure of H_2 and CO_2 on the reaction rate and catalyst stability

CO_2 hydrogenation is performed at 20 bar, 180 °C, and 120000 h^{-1}. The inlet gas mixture is modified as indicated below according to each specific study:

- Study of the Influence of the partial pressure of H_2 at a constant partial pressure of CO_2 (P_{CO2} = 4.7 bar; i.e., 23.7 vol %CO_2) (Fig S15a): The reaction composition is labelled as CO_2:H_2 (X : X) according to the CO_2:H_2 molar ratio. Thus, CO_2:H_2 (1:1) correspond to a reactant feed composition of 23.7 % CO_2, 23.7 % H_2, 43 % N_2; CO_2:H_2 (1:2) of 23.7 % CO_2, 47.5 % H_2, 28.8 % N_2; CO_2:H_2 (1:3) of 23.7 % CO_2, 71.3 % H_2, 5 % N_2 and CO_2:H_2 (1:3.6) of 23.7 % CO_2, 75.9 % H_2, 0.4 % N_2.
- Study of the influence of the partial pressure of CO_2 at a constant partial pressure of H_2 (P_{H2} =9.5 bar) (Fig S15b): The reaction composition is labelled as CO_2:H_2 (X : X) according to the CO_2:H_2 molar

ratio. Thus, CO_2:H_2 (1:5) correspond to a reactant feed composition of 9.5 % CO_2, 47.5 % H_2, 43 % N_2; CO_2:H_2 (1:2) of 23.7 % CO_2, 47.5 % H_2, 28.8 % N_2 and CO_2:H_2 (1:1) of 47.5 % CO_2, 47.5 % H_2, 5 % N_2.

These feed compositions allow variation in the partial pressure of H_2 (15.2, 14.3, 9.5, and 4.7 bar) and CO_2 (9.5, 4.7 and 1.9 bar), obtaining the intrinsic rate of methane formation in each case. The measured rates are fitter by a power law kinetic model following the Equation (4), where r_{CH4} corresponds to the intrinsic rate of methane formation, α and β correspond to the apparent reaction orders with respect to the H_2 and CO_2 partial pressures, respectively.

$$r_{CH_4} = kP_{H_2}^{\alpha}P_{CO_2}^{\beta} \#(4)$$

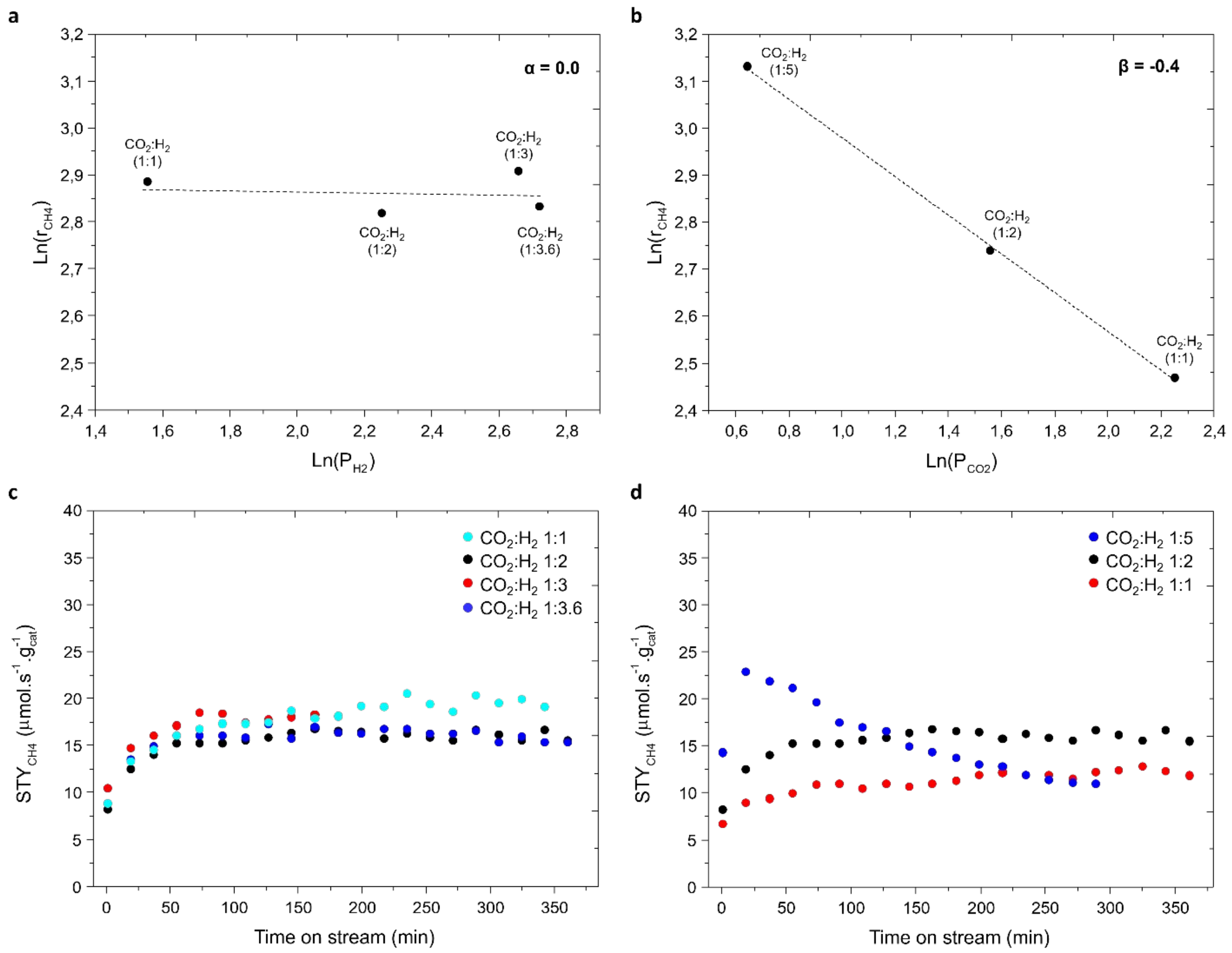

Figure S15. Reaction orders for RuO_xC_y@C catalyst at 180 °C and 20 bar. Intrinsic rates for CH_4 formation as a function of: a) the partial pressure of H_2 (at constant P_{CO2} = 4.7 bar), and b) the partial pressure of CO_2 (at constant P_{H2} = 9.5 bar). In brackets is the CO_2:H_2 molar ratio. c) Influence on the catalyst stability (i.e.,

methane production with time on stream) of the reaction composition used in panel a. Cyan line (23.7 % CO_2, 23.7 % H_2, 43 % N_2) (CO_2:H_2= 1:1), black line (23.7 % CO_2, 47.5 % H_2, 28.8 % N_2) (CO_2:H_2= 1:2), red line (23.7 % CO_2, 71.3 % H_2, 5 % N_2) (CO_2:H_2= 1:3), and blue line (23.7 % CO_2, 75.9 % H_2, 0.4 % N_2) (CO_2:H_2= 1:3.6). d) Influence on the catalyst stability (i.e., methane production with time on stream) of the reaction composition used in panel b. Blue line (9.5 % CO_2, 47.5 % H_2, 43 % N_2) (CO_2:H_2= 1:5),black line (23.7 % CO_2, 47.5 % H_2, 28.8 % N_2) (CO_2:H_2= 1:2), and red line (47.5 % CO_2, 47.5 % H_2, 5 % N_2) (CO_2:H_2= 1:1). Reaction conditions: 180 °C and GHSV 120000 h^{-1}.

The XRD pattern of used samples corresponding to the experiments displayed in Figure S16 c and d are given in Figure S16. Figure S16a shows the XRD pattern of the used samples after exposure to different partial pressures of H_2 at constant P_{CO2} = 4.7 bar, while Figure S16b shows the XRD pattern of the used samples after exposure to different partial pressures of CO_2 at constant P_{H2} = 9.5 bar. In all the cases, the XRD diffractogram patterns show a diffraction peak around 26°, which is related to the RuO_2C_y phase together with RuO_2 (peak around 28°) and Ru^0 (peak around 46°), confirming the coexistence of the three crystalline phases. However, in the experiments of Figure S16b using CO_2:H_2= 1:5, Ru^0 (peak around 46°) is predominately observed, indicating a detrimental role of high H_2 partial pressure in the stability of the catalyst.

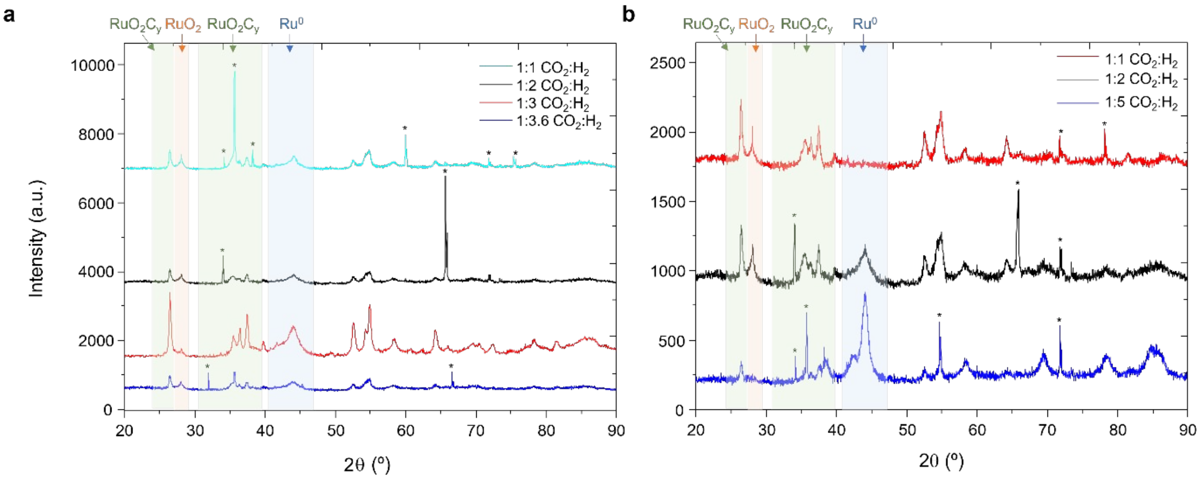

Figure S16. (a) XRD pattern of the used samples after being exposed to different partial pressures of CO_2 at constant P_{H2} = 9.5. Cyan line (23.7 % CO_2, 23.7 % H_2, 43 % N_2) (CO_2:H_2= 1:1), black line (23.7 % CO_2, 47.5 % H_2, 28.8 % N_2) (CO_2:H_2= 1:2), red line (23.7 % CO_2, 71.3 % H_2, 5 % N_2) (CO_2:H_2= 1:3), and blue line (23.7 % CO_2, 75.9 % H_2, 0.4 % N_2) (CO_2:H_2= 1:3.6). (b) XRD pattern of the samples after being exposed to different

partial pressures of H_2 at constant P_{CO2} = 4.7. Blue line (9.5 % CO_2, 47.5 % H_2, 43 % N_2) (CO_2:H_2= 1:5), black line (23.7 % CO_2, 47.5 % H_2, 28.8 % N_2) (CO_2:H_2= 1:2), and red line (47.5 % CO_2, 47.5 % H_2, 5 % N_2) (CO_2:H_2= 1:1). The asterisk corresponds to the SiC used in the catalytic test as diluent. In colour the different zones correspond to RuO_2C_y (green), RuO_2 (red), and Ru^0 (blue). For more information about the different diffraction patterns, see Figure S2 and Tables S1-S3.

1.4.4. Space velocity and its influence on the catalyst performance

The influence of the space velocity (GHSV) is studied at 20, 10 and 1 bar.

CO_2 hydrogenation at 20 bar, 180 °C, and CO_2:H_2= 1:3 (100 mL/min). In this set of experiments, the space velocity is modified by changing the mass of the catalyst, while keeping the rest of variables constant. The reaction conditions are kept for 18721 min (i.e., 13 days) in the case of 24000 h^{-1} (Figure S17, in red) and 4447 min in the case of 120000 h^{-1} (Figure S17, in black).

Figure S17 shows that, at the same reaction conditions, a lower space velocity favours the stability of the catalyst.

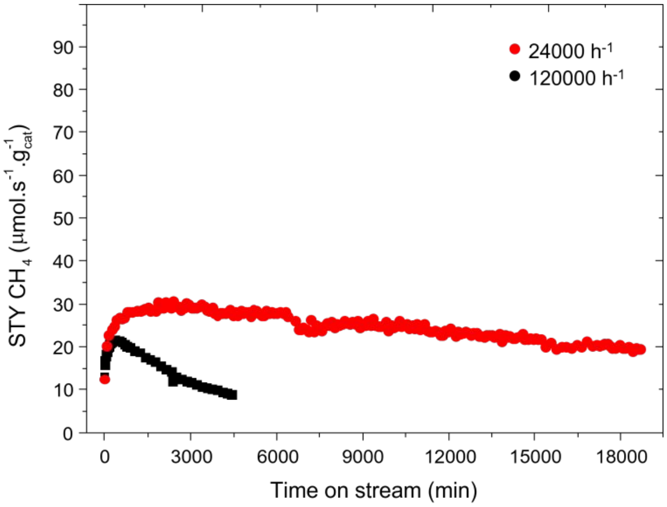

Figure S17. Influence of the gas space velocity on the RuO_xC_y@C catalytic performance at 180 °C and 20 bar. Reaction conditions: in red: 24000 h^{-1} and 100 mL/min (CO_2:H_2 1:3); in black: 120000 h^{-1} and 100 mL/min (CO_2:H_2 1:3).

CO_2 hydrogenation at 10 and 1 bar, 180 °C, and CO_2:H_2= 1:3 (100 mL/min). Similar to before the space velocity is modified by changing the mass of the catalyst, while keeping the rest of the variables constant. For the experiment at 10 bar, these conditions are kept for 9615 min for 24000 h^{-1} (Figure S18 a, in red) and 361 min in the case of 120000 h^{-1} (Figure S18 a, in black). For the experiment at 1 bar, these conditions

are kept for 3288 min for 24000 h^{-1} (Figure S18 b, in red) and 361 min in the case of 120000 h^{-1} (Figure S18 b, in black).

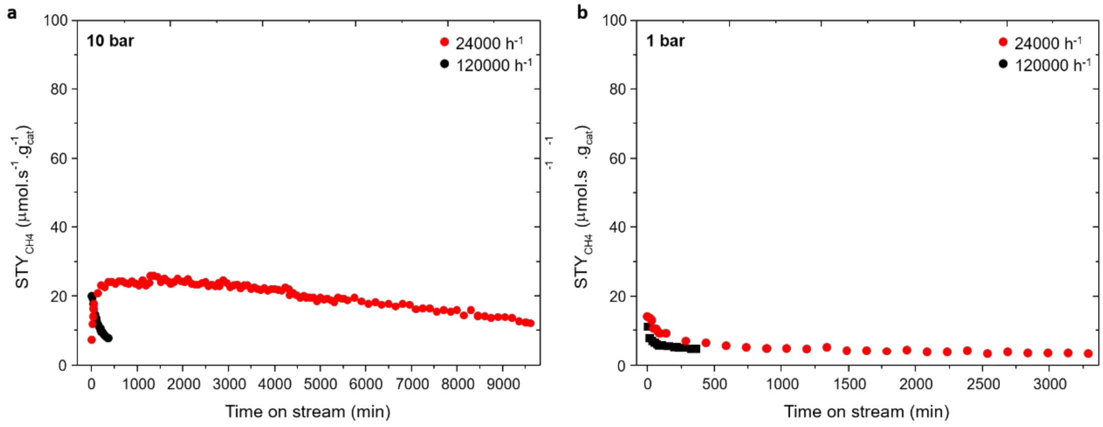

Figure S18. Influence of the gas space velocity on the RuO_xC_y@C catalytic performance at 180 °C and different pressures: (a) 10 bar and (b) 1 bar. Reaction conditions: in red: 24000 h^{-1} and 100 mL/min (CO_2:H_2 1:3; in black: 120000 h^{-1} and 100 mL/min (CO_2:H_2 1:3).

Figure S18 shows that the instability of the catalyst, when operating at 10 bar, can partially be recovered by decreasing the space velocity, whereas at 1 bar, it cannot be recovered even when operating at a lower space velocity.

1.4.5. CO_2 hydrogenation transient study

The absence of CO formation during catalytic studies generates uncertainty about whether the mechanism of CH_4 formation proceeds through CO or an associative mechanism. To better understand the intermediates involved, the hydrogenation of carbon intermediates was studied through a transient study. The experiment was carried out at 10 bar, 140 °C and GHSV = 16500 h^{-1}. Under these operational conditions, 30 % CO_2 was fed in a stream of N_2 (70 % N_2), and after 1.5 hours, the feed gas was switched to 60 % H_2 in N_2 (40 % N_2) to stimulate the hydrogenation of intermediates of carbon on the surface of the catalyst. The effluent was monitored continuously in a mass spectrometer (Balzer, QMG 220M1). The response of the signals of CO_2 (*m/z*: 44), H_2 (*m/z*: 2), H_2O (*m/z*: 18), CO (*m/z*: 28) and CH_4 (*m/z*: 15) are shown in Figure S19a.

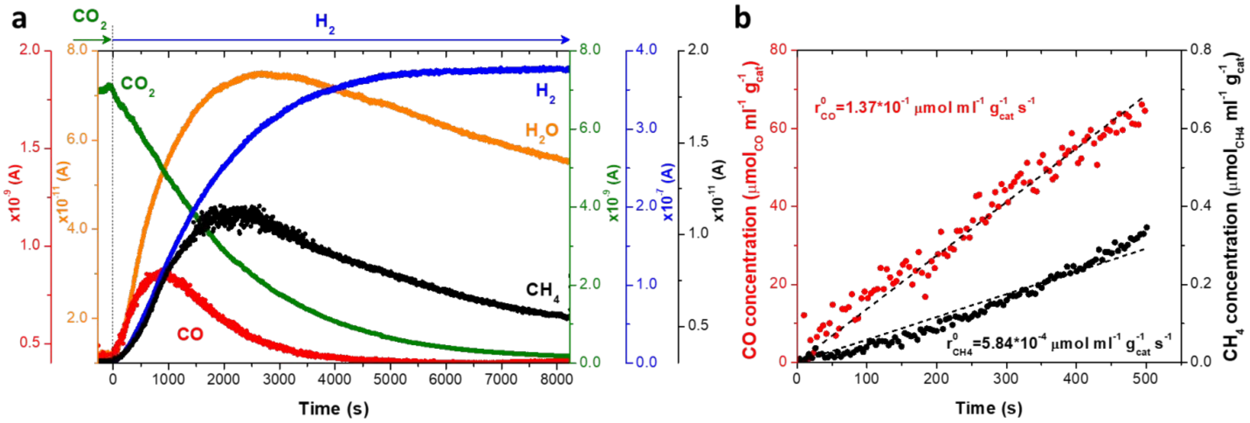

Figure S19. a) MS signal after the switch of the feed from N_2:CO_2 (2.5:1) to H_2:N_2 (2:1.5) over RuO_xC_y@C catalyst, b) Initial rate of CO and CH_4 formation after feeding H_2. Reaction conditions: 140 °C, 10 bar and 16500 h^{-1}.

The transient study shows that CO is formed in the catalyst. Furthermore, the calculation of initial rates (Figure S19b) shows that CO formation is almost immediate once the feed is switched to H_2, reaching an initial CO formation rate of 1.37x10^{-1} µmol mL^{-1} g_{cat}. Therefore, a CO_2 dissociative mechanism may be occurring. The competitive adsorption of H_2 displacing weakly adsorbed CO probably from the Ru^0 sites explains very well the results of the transient study and the apparent negative order of CO_2. On the other hand, methane formation is slower, with an initial rate of 5.84x10^{-4} µmol ml^{-1} g_{cat}^{-1} s^{-1}, typical of more complex mechanisms with several steps involved. However, the extensive tail may represent a high density of methane active sites, or a high coverage of methane formation intermediates.

To achieve a clear understanding of the role that CO plays in the reaction mechanism, additional experiments were proposed where CO is co-fed with the CO_2 reaction mixture.

1.4.6. CO_2 hydrogenation in the presence of trace amount of CO

CO is added at different concentrations, keeping the concentration of CO_2 and H_2 constant. The volume percentage used in each experiment are:

- No CO (71.3% H_2, 23.7% CO_2, and 5% N_2).
- 1.5 % CO vol (71.3% H_2, 23.7% CO_2, 1.5% CO, and 3.5% N_2).
- 3.7 % CO vol (71.3% H_2, 23.7% CO_2, 3.7% CO, and 1.2% N_2).

The reaction conditions are 180 °C, CO_2:H_2 = 1:3 (100 mL/min), and 120000 h^{-1}, and the reaction pressure varies between 1, 10 and 20 bar. The separate CO_2 and CO conversion and their associated methane production (in µmol/s·g_{cat}) at different CO percent (1.5 and 3.7 % vol) and reaction pressures are shown in Table S7.

In all cases methane is present as the main product, however, at increasing CO amount, other sub-products are formed as indicated in Figure S20.

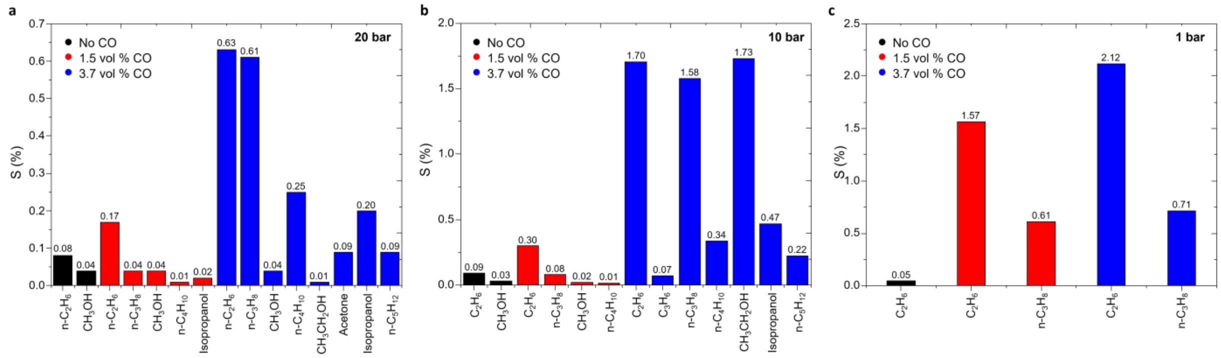

Figure S20. Product distribution at different CO concentration in the reactant feed and operating at different reaction pressures: (a) 20 bar, (b) 10 bar, and (c) 1 bar. Reaction conditions: 100 mL/min (CO_2:H_2 1:3), and 120000 h^{-1}. The black colour refers the experiments in which no CO is added, and the red and blue to the experiments in which 1.5 and 3.7 % CO are co-feed, respectively.

Table S7. Separate CO_2 and CO conversion, methane production (in brackets in μmol/s·g_{cat}) associated to the moles converted of each reactant, and methane selectivity, at different CO percent (1.5 and 3.7 % vol) and reaction pressures.

	**20 bar**			**10 bar**			**1 bar**		
	X_{CO2} **(%)** (*μmol$_{CH4}$/s·g$_{cat}$*)	X_{CO} **(%)** (*μmol$_{CH4}$/s·g$_{cat}$*)	S_{CH4} **(%)**	X_{CO2} **(%)** (*μmol$_{CH4}$/s·g$_{cat}$*)	X_{CO} **(%)** (*μmol$_{CH4}$/s·g$_{cat}$*)	S_{CH4} **(%)**	X_{CO2} **(%)** (*μmol$_{CH4}$/s·g$_{cat}$*)	X_{CO} **(%)** (*μmol$_{CH4}$/s·g$_{cat}$*)	S_{CH4} **(%)**
0% vol CO	10.0 (*17.2*)	-	99.9	7.35 (*10.6*)	-	99.9	3 (*4.7*)	-	99.9
1.5% vol CO	9.5 (*16.4*)	100 (*10.8*)	99.7	6.37 (*9.7*)	100 (*10.8*)	99.6	0 (*0.0*)	22 (*2.5*)	97.8
3.7% vol CO	3.2 (*2.2*)	100 (*26.9*)	98.1	0.00 (*0.0*)	90 (*23.6*)	93.9	0 (*0.0*)	18 (*5.3*)	97.2

The XRD pattern of the used samples after exposure to 1 and 10 bar without CO (red line in Figure 5 a,b of the main text) shows a diffraction peak around 42°, characteristic of the Ru^0. When 3.7% vol of CO is added to the reactant feed at 1 bar (Figure 5a, black line), the diffraction peak due to Ru^0 decreases, whereas the peak around 26° related to the RuO_2C_y phase, which is not present in the absence of CO under similar reaction conditions, appear. This clearly indicates a positive role of CO in stabilizing the RuO_2C_y crystalline phase. At 10 bar, the XRD diffraction pattern of the used sample by adding 3.5 % vol of CO to the reactant feed (Figure 5 b, black line) shows a decrease of the peak due to Ru^0, while the peak due to the RuO_2C_y crystalline phase remains.

Thus, in both cases, a positive effect of CO in the stabilization of the RuO_2C_y crystalline phase by suppressing their reductive degradation is observed.

2. Ru^0-RuO_xC_y@C catalysts

2.1. Synthesis of Ru^0- RuO_xC_y@C sample

Ru^0-RuO_xC_y@C sample is prepared using the synthetic procedure described in ref 10, but using another precursor for RuO_2. In this case, RuO_2 from Alfa Aesar with a particle size of 24 nm is used. In detail, the catalyst is synthesized by mixing 120 mg of glucose (Aldrich, > 99.5 %), 7 mL of miliQ water, and 100 mg of RuO_2. Then, all the chemicals are loaded into a Teflon-coated stainless-steel autoclave of 15 mL and introduced in an oven at 175 °C under static conditions for 24 h. Afterwards, the autoclave is removed from the oven and cooled to room temperature for 2 h. The black solid of the autoclave is filtrated and washed with abundant distilled water and acetone. It is then dried in an oven at 60 °C overnight.

In this synthesis, RuO_2 with lower particle size, i.e., 24 nm is used in order to favor a higher amount of Ru^0 in the final sample. Notice that the herein used hydrothermal synthesis conditions are slightly reductive.

2.2. Temperature programme reduction in H_2 (TPR-H_2)

TPR-H_2 analyses are performed on a Micromeritics Autochem 2910 instrument. Approximately 50 mg of sample is cleaned with 30 mL·min^{-1} of Ar at 25°C for 30 min. Next, a mixture of 10 vol % of H_2 in Ar (total flow 50 mL·min^{-1}) is passed through the solid while the temperature is increased up to 600 °C (10 °C·min^{-1}).

Fig. S21 shows the TPR-H_2 of RuO_2-39 nm and RuO_2-24 nm used as precursors for the RuO_xC_y@C and Ru^0-RuO_xC_y@C samples, respectively. A lower reducibility is observed on RuO_2-24 nm (peak maxima at 83 °C)

compared to RuO_2-39 nm with a peak maximum at 105°C, in line with the smaller particle size of the former (24 nm) versus the last one (39 nm).

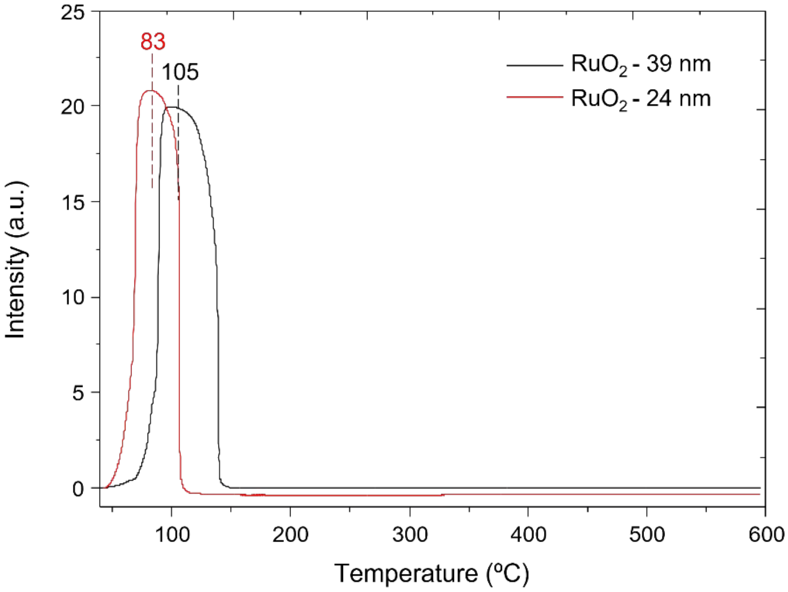

Figure S21. TPR-H_2 of the RuO_2 -39 nm (in black) and RuO_2 -24 nm (in red) samples.

2.3. Spectroscopic characterisation of the fresh and used samples at 1, 10 and 20 bar

2.3.1. X-Ray Diffraction (XRD)

Figure S22 shows the XRD of the Ru^0-RuO_xC_y@C fresh sample. According to XRD data, the composition is 55 ± 1 % wt for RuO_2C_y and 45 ± 1 % wt for Ru^0.

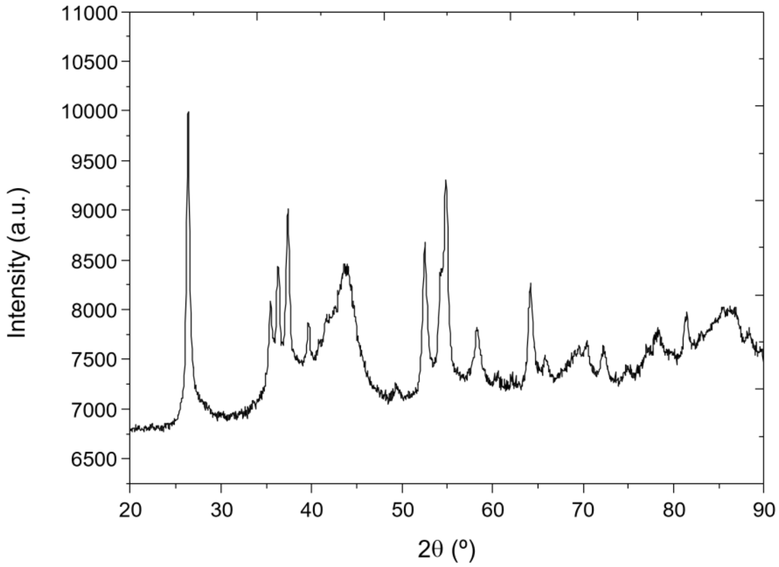

Figure S22. XRD of fresh Ru^0-RuO_xC_y@C sample.

The XRD pattern of the used Ru^0-RuO_xC_y@C samples after exposure to 1, 10 and 20 bar is shown in Figure S23. At 1 and 10 bar, Ru^0 (peak around 42 °) dominates, whereas at 20 bar, Ru^0 is less predominant.

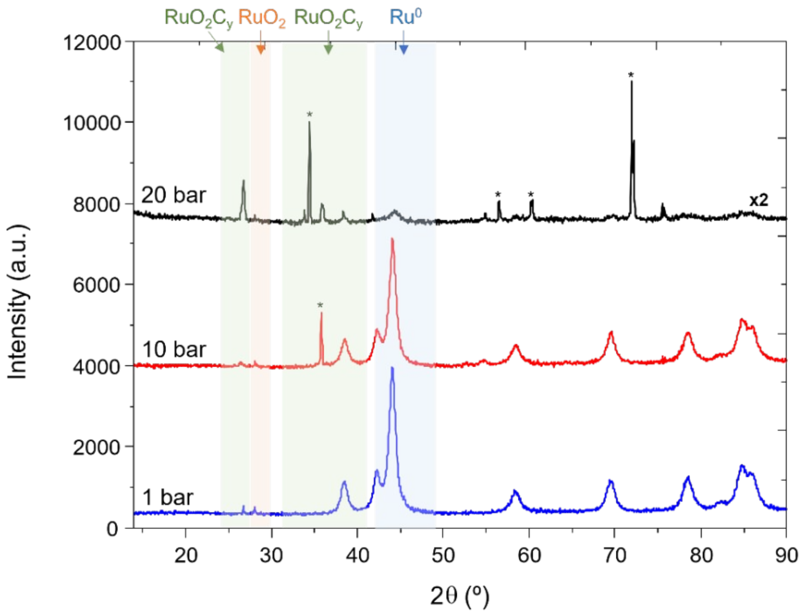

Figure S23. XRD of used Ru^0-RuO_xC_y@C samples after 1, 10 and 20 bar. The asterisk corresponds to the SiC used in the catalytic test as diluent. In colour the different zones correspond to RuO_2C_y (green), RuO_2 (red), and Ru^0 (blue). For more information about the different diffraction patterns, see Figure S2 and Tables S1-S3.

2.3.2. X-ray Absorption Spectra (XAS)

Figures S24-S26 show the XAS data for the fresh and used at 1 and 10 bar Ru^0-RuO_xC_y@C sample.

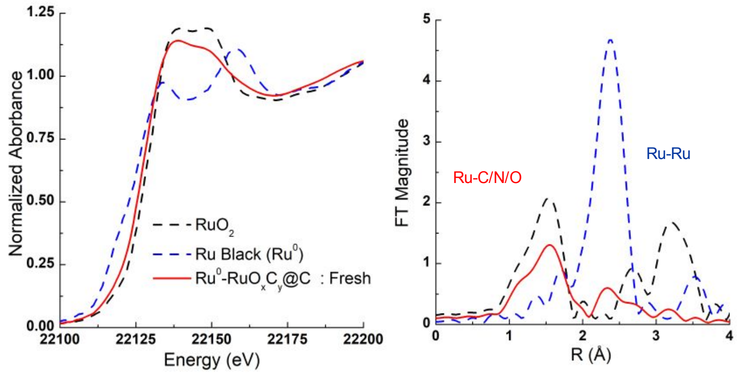

Figure S24. (Left) XANES rising edge of Ru^0- RuO_xC_y@C for fresh samples contrasted to references Ru black (Ru^0) and RuO_2. (Right): Fourier transformed k^2-weighted EXAFS spectrum with a k-range of 3-14 $Å^{-1}$ and a Hannings window; formation of Ru^0 is observed at 2.4 Å in r-space. The feature at 2.4 A in the FT spectrum shows the contribution of Ru^0, which is already present in the starting material, in line with SXRD).

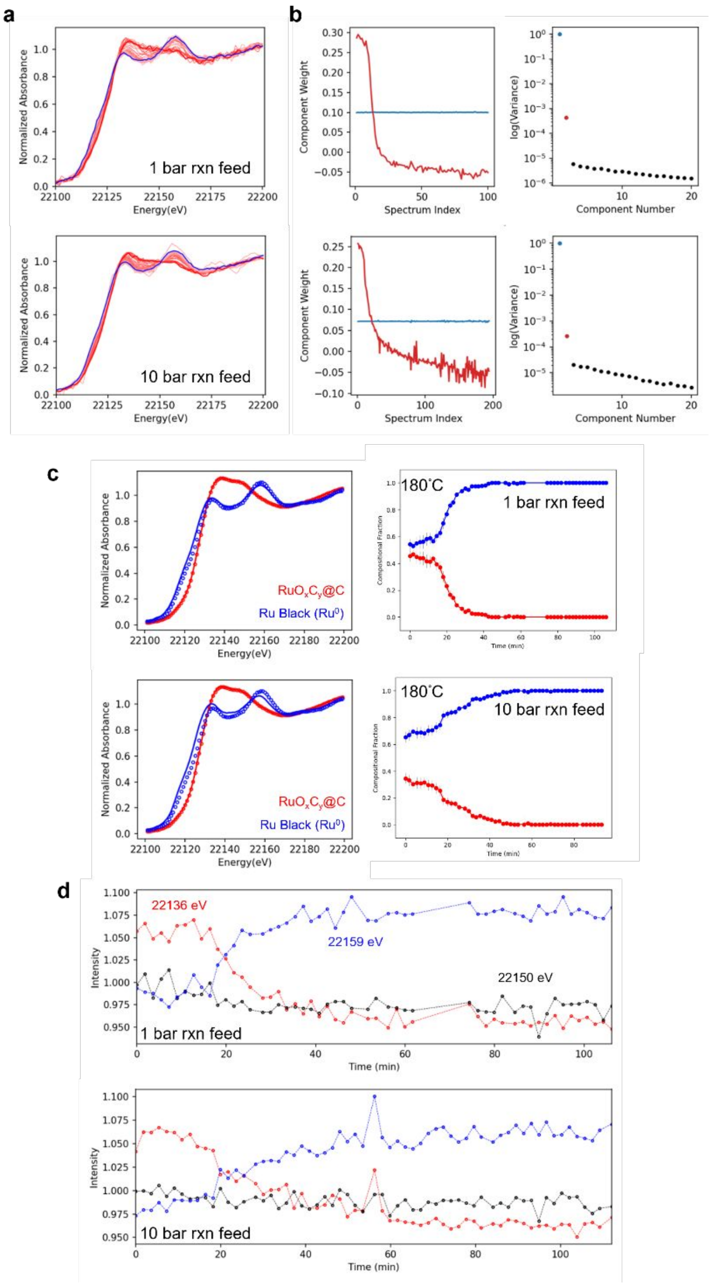

Figure S25. Operando data for Ru^0-RuO_xC_y@C under reaction conditions (CO_2+H_2) at 1 and 10 bar: (a) XANES of operando data; (b) PCA derived, eigenvectors and scree plot; (c) MCR-ALS analysis showing the input guess component spectra (dots) output determined optimized spectra (lines) and on bottom evolution of fractional contribution over time. Spectra were optimized using Ordinary Least-Squares, with the sum to 1 and non-negativity constraints for the concentrations (d) Intensity traces at 22136 eV (red), 22150 eV (black) and 22159 eV (blue) that best correlate to Ru^0- RuO_xC_y@C, RuO_2 and Ru Black, respectively.

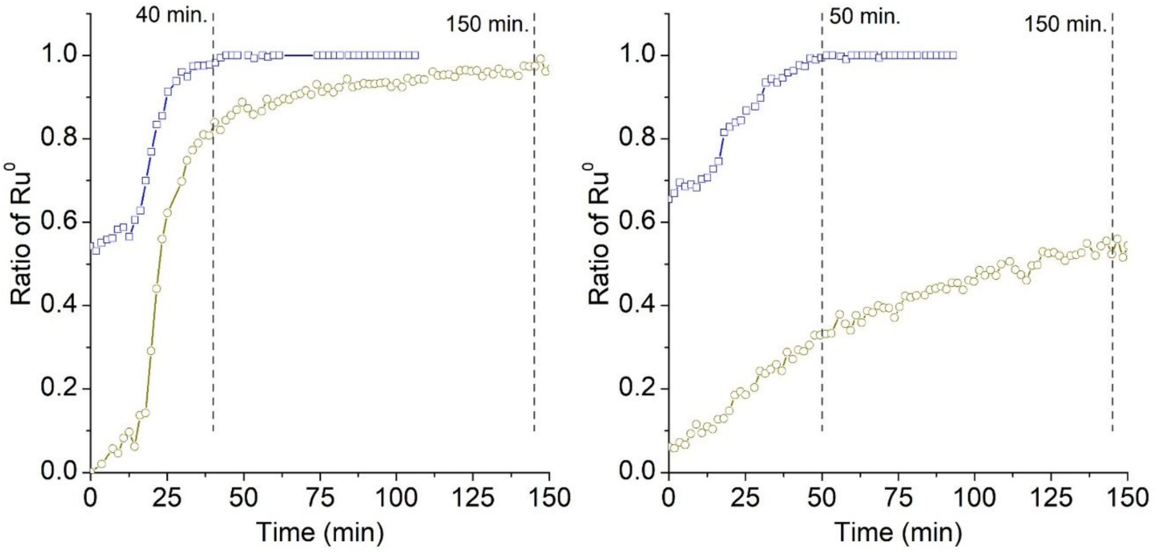

Figure S26. Formation of Ru^0 at 1 bar (left) and 10 bar (right) for Ru^0-RuO_xC_y@C (blue squares) and RuO_xC_y@C (yellow circles) under reaction conditions.

2.3.2.1. Kinetics studies of RuO_2C_y disappearance

The time evolution of the RuO_2C_y component under the reaction conditions displayed in Figure S27 are analysed in order to determine the kinetics of catalyst deactivation. The data are fitted with a linear regression to the Equations (1-3) indicated in section 1.2.2.1, which describe the different deactivation orders calculated from the general expression $-da/dt = kA^{\mathrm{d}}$, where k represents the deactivation constant and d the deactivation order (i.e., Equation 1 for $\mathrm{d} = 0$, Equation 2 for $\mathrm{d} = 1$, and Equation 3 for $\mathrm{d} = 2$) *(32)*. Figure S27 shows different fittings depending on the velocity order for the evolution of the RuO_2C_y phase to Ru^0 obtained by XAS under different reaction conditions (i.e., 10 and 1 bar). The analysed data are between ~15 min and 100 min for the experiments done at 1 bar (Fig S27 a), and between ~15 min and 90 min for the experiments at 10 bar (Fig S27 b).

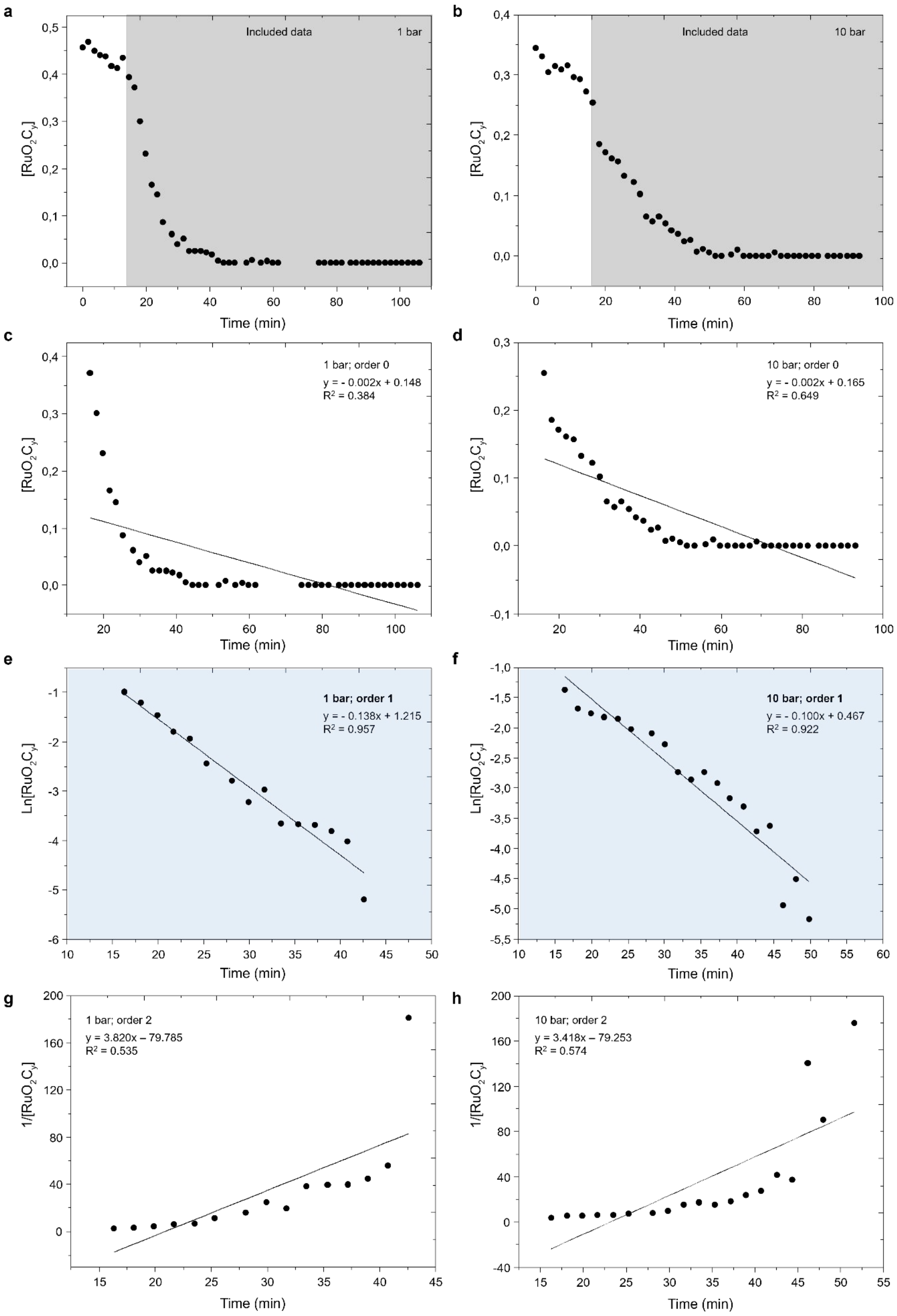
a
Included data
1 bar
[RuO2Cy]
Time (min)
b
Included data
10 bar
[RuO2Cy]
Time (min)
c
1 bar; order 0
y = - 0.002x + 0.148
R2 = 0.384
[RuO2Cy]
Time (min)
d
10 bar; order 0
y = - 0.002x + 0.165
R2 = 0.649
[RuO2Cy]
Time (min)
e
1 bar; order 1
y = - 0.138x + 1.215
R2 = 0.957
Ln[RuO2Cy]
Time (min)
f
10 bar; order 1
y = - 0.100x + 0.467
R2 = 0.922
Ln[RuO2Cy]
Time (min)
g
1 bar; order 2
y = 3.820x – 79.785
R2 = 0.535
1/[RuO2Cy]
Time (min)
h
10 bar; order 2
y = 3.418x – 79.253
R2 = 0.574
1/[RuO2Cy]
Time (min)

Figure S27. (a-c) Evolution of the RuO_2Cy phase with time at different pressures: (a) 1 and (b) 10 bar. The grey zone corresponds to the data that are used for the kinetics fittings. (c-d) lineal regression of the evolution of the RuO_2C_y phase with time at the different reaction conditions: 1 (c) and 10 bar (d), corresponding to a cero order equation. (e-f) Lineal regression of the evolution of Ln(RuO_2C_y) with time at the different reaction conditions: 1 (e) and 10 bar (f), corresponding to a one order equation. (g-h) Lineal regression of the evolution of 1/(RuO_2C_y) with time at the different reaction conditions: 1 (g) and 10 bar (h), corresponding to a second order equation. The blue graphs correspond with the better linear fitting (i.e., one order for the processes at 1 and 10 bar).

2.3.3. Synchrotron X-Ray Photoelectron Spectroscopy (XPS)

Synchrotron XPS spectra obtained of the fresh and used samples at 1, 10 and 20 bar are given in Fig. S28 and summarized in Table S8 and Figure S29. The used samples have been measured ex situ after removal from the reactor and preservation in a N_2 atmosphere.

Table S8. Synchrotron XPS of the C 1s and Ru $3d_{5/2}$ core levels on fresh and used samples after 20, 10 and 1 bar. BE (eV) of each component analyzed at two X-ray excitation energy: 500 eV (i.e., depth 1.9 nm) and 1400 eV (i.e., depth 5.6 nm). The numbers in brackets represent the total surface atomic percentage of the components.

Table S8. Synchrotron XPS of the C 1s and Ru $3d_{5/2}$ core levels on fresh and used samples after 20, 10 and 1 bar. BE (eV) of each component analyzed at two X-ray excitation energy: 500 eV (i.e., depth 1.9 nm) and 1400 eV (i.e., depth 5.6 nm). The numbers in brackets represent the total surface atomic percentage of the components.

Sample	Depth	C 1s				Ru 3d $_{5/2}$					C : Ru (atom ratio)
		C-C,C-H	C-OH	RHC=O	RCOOH/ R-O-C(=O)-O-R'	Ru^0	RuO_2C_y	Ru ^{IV}	s.Ru ^{IV}	Ru ^{VI}	
Ru^0-RuO_xC_y@C	1.9 nm	284.5 *(15.5)*	285.2 *(47.4)*	-	288.5 *(10.4)*	-	**280.3** *(0.9)*	280.8 *(13.1)*	282.7 *(6.3)*	281.2 *(6.3)*	73.3 : 26.7
	5.6 nm	284.5 *(39.0)*	286.2 *(13.0)*	-	288.4 *(20.2)*	-	280.6 *(3.0)*	281.1 *(14.4)*	282.9 *(5.4)*	281.5 *(5.0)*	72.2 : 27.7
Ru^0-RuO_xC_y@C 20 bar	1.9 nm	284.5 *(66.7)*	286.7 *(8.7)*	-	288.4 *(3.2)*	279.7 *(3.8)*	280.2 *(9.6)*	280.6 *(3.3)*	282.5 *(2.1)*	281.7 *(2.6)*	78.5 : 21.5
	5.6 nm	284.5 *(73.9)*	-	-	-	279.8 *(12.3)*	280.3 *(8.6)*	280.8 *(2.5)*	282.7 *(0.9)*	281.9 (1.6)	73.9 : 26.0
Ru^0-RuO_xC_y@C 10 bar	1.9 nm	284.5 *(46.4)*	-	287.0 *(11.6)*	-	279.7 *(12.2)*	280.2 *(20.9)*	280.6 *(3.2)*	282.0 *(5.6)*	-	57.9 : 41.9
	5.6 nm	284.5 *(38.6)*	-	-	-	279.7 *(36.2)*	280.1 *(18.6)*	280.6 *(1.65)*	281.9 (4.8)	-	38.6 : 61.3
Ru^0-RuO_xC_y@C 1 bar	1.9 nm	284.5 *(43.8)*	-	-	-	279.6 *(24.2)*	**280.1** *(26.5)*	281.9 *(5.4)*	-	-	43.8 : 56.1
	5.6 nm	284.5 *(43.2)*	-	-	-	279.5 *(37.5)*	279.8 *(18.3)*	281.5 *(0.9)*	-	-	43.2 : 56.7

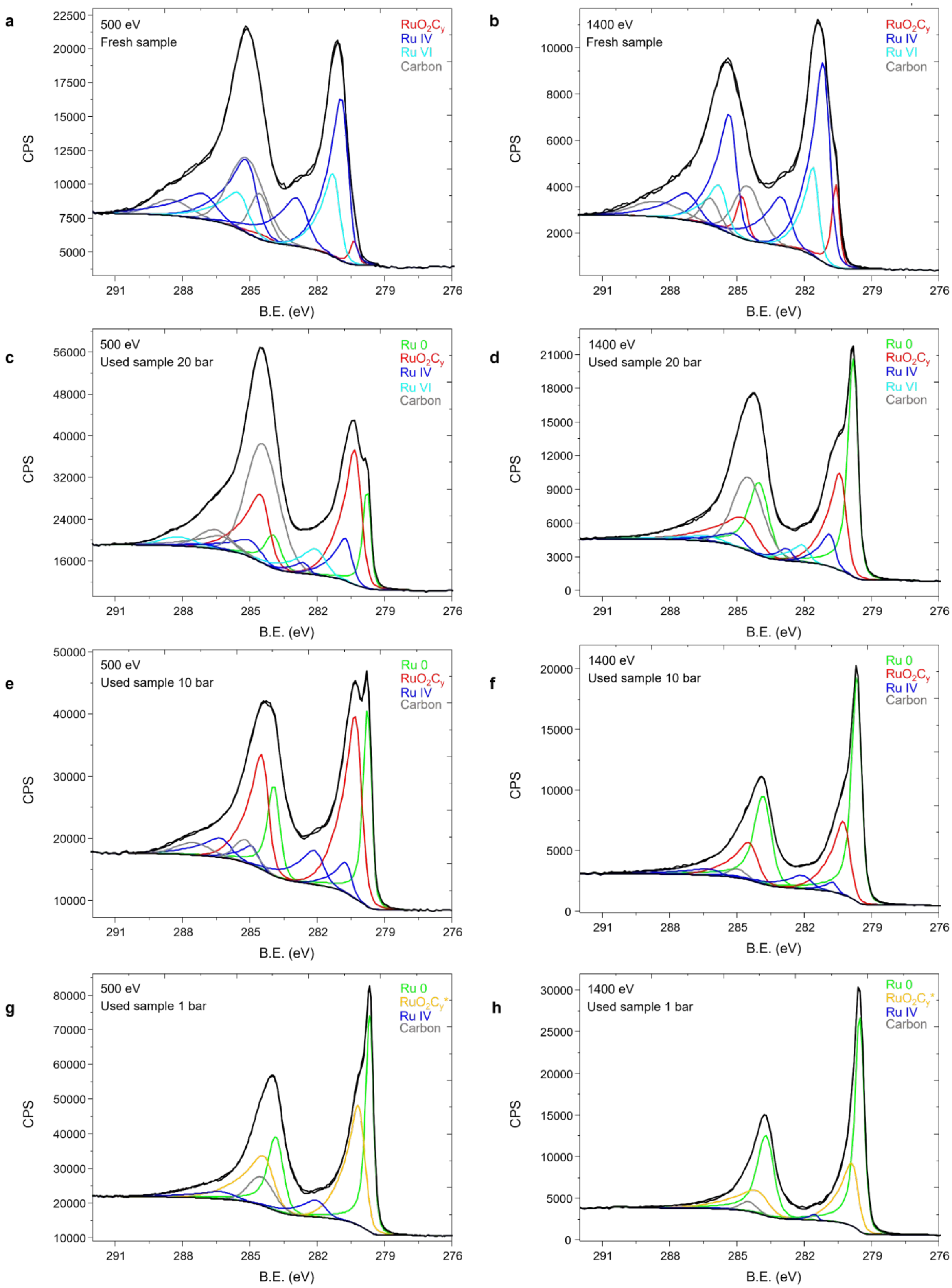
a
500 eV
Fresh sample
RuO_2C_y
Ru IV
Ru VI
Carbon
b
1400 eV
Fresh sample
RuO_2C_y
Ru IV
Ru VI
Carbon
c
500 eV
Used sample 20 bar
Ru 0
RuO_2C_y
Ru IV
Ru VI
Carbon
d
1400 eV
Used sample 20 bar
Ru 0
RuO_2C_y
Ru IV
Ru VI
Carbon
e
500 eV
Used sample 10 bar
Ru 0
RuO_2C_y
Ru IV
Carbon
f
1400 eV
Used sample 10 bar
Ru 0
RuO_2C_y
Ru IV
Carbon
g
500 eV
Used sample 1 bar
Ru 0
RuO_2C_y*
Ru IV
Carbon
h
1400 eV
Used sample 1 bar
Ru 0
RuO_2C_y*
Ru IV
Carbon
CPS
B.E. (eV)

Figure S28. Synchrotron XPS of the C 1s and Ru 3d core levels on fresh (a, b) and used samples after 20 (c,d), 10 (e,f) and 1 (g,h) bar. X-ray excitation energy is 500 eV (i.e., depth 1.9 nm) for the left spectra, and 1400 eV (i.e., depth 5.6 nm) for the right spectra. Carbon in grey, ruthenium oxy-carbonate (labelled as RuO_2C_y (in red), a degraded RuO_2C_y phase due to carbon or oxygen loss (labelled as RuO_2C_y*, in orange), metallic ruthenium (labelled as Ru^0, in green), and oxidized ruthenium (labelled as Ru IV and Ru VI in dark blue and cyan, respectively).

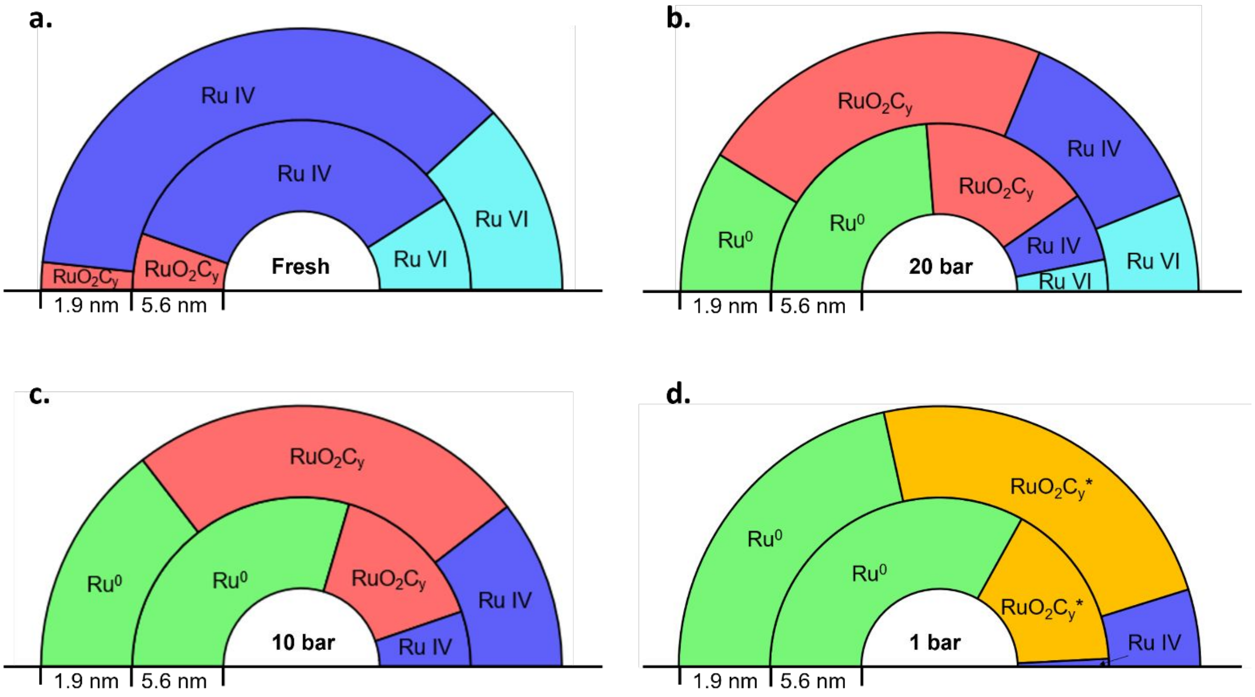

Fig. S29. Atomic fraction of the different ruthenium species in: (a) fresh, (b) used sample at 20 bar, (c) used sample at 10 bar, and (d) used sample at 1 bar. The semicircles show the atomic fraction of ruthenium oxy-carbonate (labelled as RuO_2C_y (in red), a degraded RuO_2C_y phase due to carbon or oxygen loss, labelled as RuO_2C_y* (in orange), metallic ruthenium (labelled as Ru^0, in green), and oxidized ruthenium (labelled as Ru IV and Ru VI in dark blue and cyan, respectively). The outer semicircle corresponds to the atomic fraction obtained at 1.9 nm depth (hν = 500 eV). The inner semicircle corresponds to the atomic fraction obtained at 5.6 nm depth (hν = 1400 eV).

3. Ru^0-RuO_xC_y@C-200

3.1 Synthesis of Ru^0- RuO_xC_y@C-200 sample

Ru^0-RuO_xC_y@C-200 sample is prepared using the synthetic procedure described in ref 10, but using RuO_2 from Alfa Aesar (24 nm) and modifying the synthesis temperature. In detail, the catalyst is synthesized by mixing 120 mg of glucose (Aldrich, > 99.5 %), 7 mL of miliQ water, and 100 mg of RuO_2 . Then, all the chemicals are loaded into a Teflon-coated stainless-steel autoclave of 15 mL and introduced in an oven at 200 °C under static conditions for 24 h. Afterwards, the autoclave is removed from the oven and cooled to room temperature for 2 h. The black solid of the autoclave is filtrated and washed with abundant distilled water and acetone. It is then dried in an oven at 60 °C overnight.

The synthesis temperature was increased in order to increase the fraction of Ru^0, which is confirmed in the XRD pattern of Figure S30.

3.2. XRD characterisation of the fresh sample

Figure 30 shows the XRD pattern of RuO_xC_y@C, Ru^0-RuO_xC_y@C and Ru^0-RuO_xC_y@C -200. As indicated in Table S9, a higher fraction of Ru^0 is observed in the Ru^0-RuO_xC_y@C-200 sample.

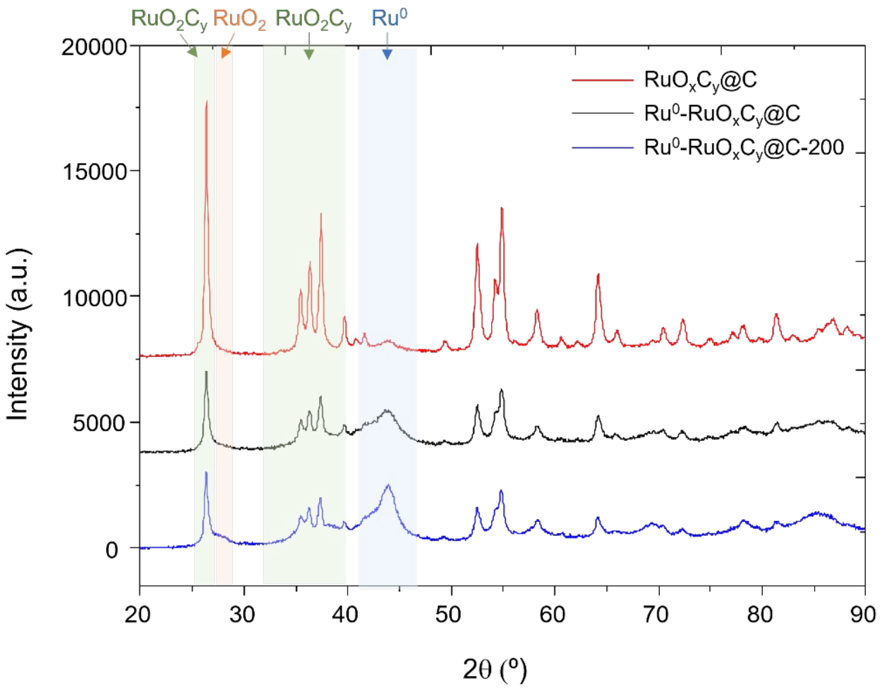

Figure S30. XRD of RuO_xC_y@C (in red), Ru^0-RuO_xC_y@C (in black), and Ru^0-RuO_xC_y@C-200 (in blue) samples. In colour the different zones correspond to RuO_2C_y (green), RuO_2 (red), and Ru^0 (blue). For more information about the different diffraction patterns, see Figure S2 and Tables S1-S3.

Table S9. Composition of the fresh RuO_xC_y@C , Ru^0-RuO_xC_y@C, and Ru^0-RuO_xC_y@C-200 samples according to SXRD data.

Sample	RuO_2C_y (%)	Ru^0 (%)
RuO_xC_y@C	90 ± 7	10 ± 5
Ru^0-RuO_xC_y@C	55 ± 7	45 ± 5
Ru^0-RuO_xC_y@C-200	50 ± 7	50 ± 5

4. Hydrogen/Deuterium (H_2/D_2) exchange experiments

H_2/D_2 exchange experiments are done using a jacket quartz flow reactor with a recirculating cooling bath that operates between 0 and 25 °C and is connected to a mass spectrometer (Balzer QMG 220M1) to analyze H_2 (m/z=2), D_2 (m/z=4), HD (m/z=3), and Ar (m/z=40).

Before the experiment, RuO_xC_y@C and Ru^0- RuO_xC_y@C samples are in situ activated in Ar (18 mL/min) for 30 minutes at room temperature to remove air from the reactor. In addition, Ru on C (5%wt Ru, Acros Organics), used as the reference sample, is in situ activated for two hours at 280 °C (10°C/min) in 25 mL/min H_2 flow.

In both cases, after sample pre-activation the temperature was lowered to 0°C in Ar flow, and once stabilized at that temperature, the Ar gas feed is changed to the isotopic mixture (4 mL min^{-1} H_2, 4 mL min^{-1} D_2, and 18 mL min^{-1} Ar). Then, the temperature is gradually raised up to 25 °C in steps of 5 °C.

In Table S10, the intensity of the m/z = 3 mass signal (in mA) of the HD component at different temperatures (from 0 to 25 °C), for both RuO_xC_y@C and Ru^0-RuO_xC_y@C, and the reference (Ru on C) sample, is given.

Table S10. Ion current values (mA) associated to the HD component (m/z = 3) at the different temperatures for RuO_xC_y@C, Ru^0-RuO_xC_y@C and the reference (Ru on C).

	HD (mA)		
T (°C)	Ru on C	RuO_xC_y@C	Ru^0-RuO_xC_y@C
0	1.03E-09	-	6.76E-11
5	1.07E-09	1.24E-11	7.30E-11
10	1.11E-09	1.43E-11	7.55E-11
15	1.13E-09	1.59E-11	7.84E-11
20	1.14E-09	1.69E-11	8.11E-11
25	1.16E-09	1.79E-11	8.27E-11

Fig. S31 shows the Napierian logarithmic of the evolution of the HD formation normalized to the H_2 (Ln(HD/H_2)) versus the inverse of the temperature (1/T). A straight line is observed for all the samples. The apparent activation energies can be found in Table S11 and the normalized H_2-D_2 isotopic exchange (HD/H_2 of HD) calculated at 25°C.

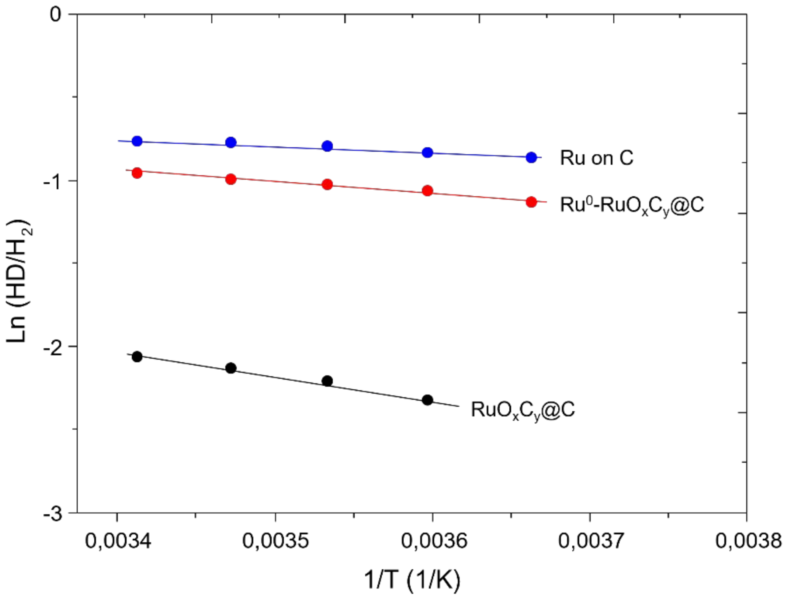

Fig. S31. Ln (HD/H_2) versus 1/T for the RuO_xC_y@C, Ru^0-RuO_xC_y@C, and reference (Ru on C) samples.

Table S11. Apparent activation energies of HD formation and normalized H_2-D_2 isotopic exchange (HD/H_2) were calculated at 25°C for the reference (Ru on C) and fresh samples (RuO_xC_y@C and Ru^0-RuO_xC_y@C).

Sample	E_{app} (kcal/mol)	HD/H_2
Ru on C	0.74 ± 0.2	0.94
RuO_xC_y@C	2.58 ± 0.2	0.27
Ru^0-RuO_xC_y@C	1.28 ± 0.2	0.78

According to Table S11, the apparent energy of RuO_xC_y@C is almost two times bigger than the others, which can be associated with the lower activity of the sample for H_2 activation. In contrast, Ru^0-RuO_xC_y@C and the Ru on C show higher activity for H_2 activation and a higher HD formation.

5. References

25. Fauth F., et al. The crystallography stations at the Alba synchrotron. Eur. Phys. J. Plus, **130**, 160 (2015).
26. Simonelli L., et al. CLÆSS: The hard X-ray absorption beamline of the ALBA CELLS synchrotron. Cogent Phys., **3**,1231987 (2016).
27. Ravel, B., and Newville, M. ATHENA, ARTEMIS, HEPHAESTUS: data analysis for X-ray absorption spectroscopy using IFEFFIT. J. Synchrotron Radiat., **12**, 537-541 (2005).
28. Newville, M. Larch: An Analysis Package for XAFS and Related Spectroscopies. J. Phys. Conf. Ser., 430 (2013).
29. Camp Jr., C. H. pyMCR: A Python Library for Multivariate Curve Resolution Analysis with Alternating Regression (MCR-AR). J. Res. Natl. Inst. Stand. Technol., **124**, 1-10 (2019).
30. Newville, M. EXAFS analysis using FEFF and FEFFI. J. Synchrotron Radiat., **8**, 96-100 (2001).
31. Rehr, J. J. and Albers, R.C. Theoretical approaches to x-ray absorption fine structure. Rev. Mod. Phys, **72**, 621-654 (2000).
32. Levenspiel, O. Ingeniería de las reacciones químicas. 2nd edn. Wiley (1990).
33. Tanuma, S., Powell, C. J. and Penn, D. R. Calculations of electron inelastic mean free paths. V. Data for 14 organic compounds over the 50–2000 eV range. Surf. Interf. Anal. **21**, 165–176 (1994).

34. Shinotsuka, H., Tanuma, S., Powell, C. J. and Penn, D. R. Calculations of electron inelastic mean free paths. X. Data for 41 elemental solids over the 50 eV to 200 keV range with the relativistic full Penn algorithm. Surf. Interf. Anal. **47**, 871–888 (2015).
35. Morgan, D. J. Resolving ruthenium: XPS studies of common ruthenium materials. Surf. Interf. Anal. **47**, 1072–1079 (2015).
36. Yeh, J. J. and Lindau, I. Atomic subshell photoionization cross-sections and asymmetry parameters: $1 \leq Z \leq 103$. Atomic Data Nucl. Data Tables **32**, 1–155 (1985).
37. Rolison, D.R., Hagans, P. L., Swider, K.E. and Long, J.W. Role of Hydrous Ruthenium Oxide in Pt−Ru Direct Methanol Fuel Cell Anode Electrocatalysts: The Importance of Mixed Electron/Proton Conductivity. Langmuir, **15**, 774–779 (1999).
38. Gaur, S. et al. CO2 Reforming of CH4 over Ru-Substituted Pyrochlore Catalysts: Effects of Temperature and Reactant Feed Ratio. Energy Fuels, **26**, 1989−1998 (2012).
39. Gonzalez-Elope, A.R., Espinos, J.P., Femandez, A. and Munuera, G. XPS study of the surface carbonation/hydroxylation state of metal oxides. Appl. Surf. Sci., **45**, 103-108 (1990).
40. Roy, P., Rouzières, M., Qi, Z. and Chubar, O. The AILES Infrared Beamline on the third generation Synchrotron Radiation Facility SOLEIL. Infrared Phys.Technol., **49**, 139-146 (2006).
41. Khorshidi, A., and Sadegh, N. Application of RuO_2 Nanoparticles as Catalyst in Preparation of Indolo[3,2-a]Carbazoles. J. Clust. Sci., **27**, 1923–1932 (2016).
